# Music viewed by its entropy content: A novel window for comparative analysis

**DOI:** 10.1371/journal.pone.0185757

**Published:** 2017-10-17

**Authors:** Gerardo Febres, Klaus Jaffe

**Affiliations:** 1 Departamento de Procesos y Sistemas, Universidad Simón Bolívar, Sartenejas, Baruta, Miranda, Venezuela; 2 Laboratorio de Evolución, Universidad Simón Bolívar, Sartenejas, Baruta, Miranda, Venezuela; University of Campinas, BRAZIL

## Abstract

Polyphonic music files were analyzed using the set of symbols that produced the Minimal Entropy Description, which we call the Fundamental Scale. This allowed us to create a novel space to represent music pieces by developing: (a) a method to adjust a textual description from its original scale of observation to an arbitrarily selected scale, (b) a method to model the structure of any textual description based on the shape of the symbol frequency profiles, and (c) the concept of higher order entropy as the entropy associated with the deviations of a frequency-ranked symbol profile from a perfect Zipfian profile. We call this diversity index the ‘2nd Order Entropy’. Applying these methods to a variety of musical pieces showed how the space of ‘symbolic specific diversity-entropy’ and that of ‘2nd order entropy’ captures characteristics that are unique to each music type, style, composer and genre. Some clustering of these properties around each musical category is shown. These methods allow us to visualize a historic trajectory of academic music across this space, from medieval to contemporary academic music. We show that the description of musical structures using entropy, symbol frequency profiles and specific symbolic diversity allows us to characterize traditional and popular expressions of music. These classification techniques promise to be useful in other disciplines for pattern recognition and machine learning.

## Introduction

Understanding the structures underlying music is an old restlessness, always present among researchers. During the 1950’s, Meyer[1] and, more recently, Huron [2] linked musical structure to our emotions and expectations. Their description of musical structure and its influence on our emotions is based on considerations of explicit musical language, as written on the music sheet. By using other analytical resources, a group of researchers, Mavromatis [3] among them, offer models for the construction of melodies that assume that a Markovian process is behind each specific style of melody. These models, based on Finite State Machines (FSMs), generalized in stochastic terms by a Hidden Markov Model (HMM) [4], are able to produce melodies that fit within a certain music style after being properly trained. Extending the HMM to include harmonies requires the identification of an inconveniently large number of states. As an alternative method, Rohrmeier [5] proposes a system of grammar rules to model harmonic progressions, an important extension of Lerdahl and Jackendorf’s [6] previous work and their Generative Theory of Tonal Music (GTTM).

We all share the intuitive idea of music as a flow of ordered sound waves. Formally, the presence of order in music was studied by Leonard Meyer [1], who pioneered the analysis of music as a phenomenon capable of creating emotions. Meyer analyzed in depth the expectancy experienced by the listener. In his explanations, Meyer used musical concepts and technical notations, which are difficult to represent in quantitative mathematical terms. But the idea of music as a means to create specific sensations such as tension, sadness, euphoria, happiness, rest and completeness is present throughout his study. Meyer described the emotions caused by music as the result of the interaction between the sound patterns perceived and the brain. In his words [1]: “The mind, for example, expects structural gaps to be filled; but what constitutes such a gap depends upon what constitutes completeness within a particular musical style system. Musical language, like verbal language, is heuristic in the sense “that its forms predetermine for us certain modes of observation and interpretation.”† Thus the expectations which result from the nature of human mental processes are always conditioned by the possibilities and probabilities inherent in the materials and their organization as presented in a particular musical style.” († Edward Sapir, “Language,” Encyclopedia of the Social Sciences, IX (New York: Macmillan Co., 1934), 157.).

Meyer’s reference to conditional probabilities implies the possibility of capturing some of the essence of musical style by observing the values of entropy associated with each music style. However, the style of music has proved to be a difficult concept to address. As occurs with other types of languages, style is a way of classifying specific musical pieces. The determination of the style is based on characteristics describing the music, the time when it was composed, and the geographical context. Some researchers have set a style framework for music by quantifying these characteristics. In 1997, R. Dannenberg, B. Thom and D. Watson [7] produced readable Musical Instrument Digital Interface (MIDI) files by recording 10-second-long trumpet performances. Dannenberg et al. used neural networks to classify the style of each recorded performance according to several features of music. In 2004, P. J. Ponce de León and J. M. Iñesta [8] measured the pitch, note duration, silence duration, pitch interval, non-diatonic notes, syncopation, and other music components to build statistical characterizations of Jazz and Classical melody pieces. Perez-Sancho, J. M. Inesta and J. Calera-Ruiz [9] approached the same problem by categorizing the texts of MIDI files. They extracted the melodies from the MIDI files and segmented the resulting texts into sequences of characters representing different lengths of music beats. In 2004, P. van Kranenburg and E. Backer [10] studied music styles starting from certain music properties. They included the entropy of some parameters as properties. These studies indicate that it is possible to recognize properties related to the musical style in an automated fashion, but none fulfills the required generality to be considered a true style recognizer. Musical style is simply too fuzzy a concept to serve as a quantitative reference framework useful for classifying, with a single value, something as complex as music.

A different approach sees music as a recursively nested group of structures (Rohrmeier [11]). Even considering just melody, music consists of kinds of fractal structures that make any attempt at its analysis a dauntingly complex task. Attempting to model polyphonic music ‘amplifies’ these difficulties to such an extent that Rohrmeier [11] considers the analysis of the structures of polyphonic music a practically impossible task.

In studies where the focus is on the music sheet, the analysis is limited to the music as the composer intended it to sound—instruments, rhythms and tempo, scales, note pitches, keys, chords, temperament, volume, etc.—but leaving out of the assessment many other effects of real music that are present when it is performed with musical instruments. This study, in contrast, is performed with the recording of sounds as expressed in computerized music files. Subtleties such as the effects of relative position of the instruments, their timbre, syncopation, mistuning, the performer's style and even errors are represented in these files to some degree, depending on the recording quality and resolution.

Other studies are devoted to detect relationships among some characteristics of musical pieces and the melody expressed as a sequence of notes. As one of those characterizations, Simonton [12] defined musical originality as inversely proportional to the number of times a specific pitch change is found within the first six notes of a piece. He evaluated the evolution of this conception of originality for more than 15600 pieces composed in a time span of more than 500 years. Simonton represented his findings as a polynomial which fluctuates several times within the period studied. Recently Hass [13] took the measurement of originality as defined by Simonton and applied it within the scope of certain geographical regions, the composer’s fame and the composer’s life span itself.

Temperley [14–16] has worked on music models based on the conditional probability of musical patterns. Temperly considers music as a stream of notes, each one defined as the concurrence of metrical structure, harmonic structure and stream structure. Despite Temperley’s models consider a broad spectrum of musical elements, the data input used for his models is based on the music sheet, thus some previous interpretation and transcription is required to feed the computer.

Some researchers have provided other useful schemas of the structures underlying music. In 2006, Mavromatis [17] presented models of Greek Chants depicting the melodic component of music as a process dominated by Markov chains. Later, in 2011, Rohrmeier [5] argued that that Markovian processes are too limited to properly model the complexity that arises when harmonies are added to melody. Rohrmeier proposes a Generative Theory of Tonal Harmony (GTTH) [5] as a set of recursive rules based on the Chomskian grammar and on the Generative Theory of Tonal Music (GTTM) by Lerdahl and Jackendorf [6]. Both branches of study—music as a phenomenon governed by Markovian processes and the recursive context-free rules to model harmonies—are developed for music as it is written on the music sheet, that is, music as an abstract entity represented by a set of meaningful symbols written on the music sheet, which are supposed to represent the sonic effects intended by the composer. As Rohrmeier points out in one of his notes in 2012 [18], even within this conception of music—its description on the music sheet, which is simpler than actual recorded sounds—GTTH does not suffice to properly model polyphonic music. In 2009, Mavromatis [19] suggested the application of the Minimal Description Length Principle (MDL) as an alternative to the Markovian models of melodies, and he explained why MDL should be a powerful tool to describe music. Nevertheless, he asserts that these advantages are subject to the huge computational complexity foreseen of the algorithms associated with this type of analysis.

Even for the most intricate pieces of music, the music sheet is rather simple when compared with the actual music and with the recorded file that can be reproduced—the sounds we hear. The quantitative analysis of music is even more demanding if polyphonic music is the subject of study. Polyphony adds more dimensions to an already nearly unmanageable problem. To deal with polyphonic music, Cox [20] measured the entropy of the sound for each time beat. Cox represents his results in two time-dependent entropy profiles: one for pitch and another for rhythm. Polyphonic music can be described as the superposition of many monophonic sound streams. The result is an overwhelmingly large number of combinations of sound frequencies. Luckily, all these sound streams are synchronized in time, and therefore, their recording in a file leads to a one-dimensional text where certain character sequences may form patterns that represent the musical elements contained in the text file. It is worthwhile to mention the branch of research related to the development of methods for the automatic transcription polyphonic music directly from sound. This is a promising area of study with important practical applications. Two Ph.D. Thesis by Klapuri [21] and Chai [22] can be mentioned as sources of information in this regard.

In an independent line of inquiry, Febres and Jaffe [23] presented the Fundamental Scale Algorithm (FSA). At issue is a method based on the MDL Principle that is applicable not only to music but also to most problems concerning the recognition of patterns in a large string of written symbols. The FSA is capable of unveiling the ‘dominant’ symbols of a description. In the present work, we apply the FSA to 455 MIDI files containing academic, traditional, and popular music. For each piece, the Fundamental Symbols—the set of symbols leading to the description of minimal symbolic entropy—is determined and the symbol frequency profiles built. To compare the shapes of profiles based on different numbers of symbols, the Scale Downgrading method is devised and presented. Additionally, a measure of Higher Order Entropy and a method for its calculation is proposed. We use these methods to represent different types of MIDI music in an entropy-diversity space. The dependence between the type of music and the selected representation space is analyzed.

## Methods

We take a sample of more than 450 MIDI pieces and apply several techniques to investigate ways to quantitatively classify music. The study uses the actual music files as the observed object. We do not use the music sheet as a description of each piece. Instead, we use the computer file produced after a performance recording or any other means of music production. Then, we obtain what we call the Fundamental Symbols of each music piece. The Fundamental Symbols are the sequences of characters forming symbols for which the corresponding entropy is minimal. At this point, we can compare music pieces by considering already available parameters, such as symbolic diversity and entropy. We go further and use the Fundamental Symbols to build symbol frequency profiles that offer a graphically shaped model of each music piece. These graphical models can be visually compared with one another. They can also be built for groups of musical pieces belonging, for example, to composers or to specific styles or periods of music, enabling us to compare among properly defined groups of pieces sharing certain conditions. To quantify these comparisons, we depict the frequency profiles using the same number of dots. We do this by applying a method we call Scale Downgrading. The comparison of two profiles having the same number of points is performed by computing the Euclidean distance separating both profiles.

We perform statistical tests to confirm the capacity of the method to differentiate certain genres and styles of music from others. The following sections describe in further detail the components of these methods. Additionally, we inspect the representations of music pieces, composers and genres in a 3-dimensional space formed by the specific diversity, entropy and second order entropy, which we define in the sections below.

### Language recognition, diversity and entropy

In this study, we propose a radically different method for studying the structure of music. Instead of analyzing the symbols written on the music sheet, we look at the sound recorded from actual performances by reading the text associated with the computerized file that contains the recording (the same as reading the text of an.MP3,.MP4 or.VAW file) or the file containing the synthetized version of the original recording (as with a MIDI file). To do this, we inspect the sequence of characters of the computerized files viewed as texts. Even for short files, this is not a simple task. A music file read as a text is a long sequence of characters that does not exhibit recognizable patterns, resulting in a code that is extremely difficult to interpret. Not knowing the rules of a grammar system, it is not possible to decide a priori how to recognize the symbols needed to interpret the description. There are no words in the sense we are used to, and the characters we see do not have any meaning to us. We cannot even be sure about the meaning of the space character “”. Thus, music files contain character strings to represent sounds according to the coding system used and the selected discretization level. But, as opposed to natural language text files, the music files do not show words or symbols that we humans can recognize without the help of some decoding device. Therefore, to find some order within these symbols—sequences of characters—that are camouflaged by the surrounding text, we consider the entropy of each possible set of symbols, that is, each possible way of reading the same written message. Given a space containing several types of elements, entropy is a property associated to the probability of finding each type of element, that is, the probability distribution associated with the set of elements classified by type. Referring to these elements as symbols, we can think of a message as a long sequence of symbols forming patterns. We may or may not recognize them, but those patterns are always there. In fact, there is always more than one possible pattern. Considering one or another pattern depends on the observer’s choice. Loosely speaking, the number of ways these symbols can be organized to form different patterns is an indication of the number of meanings we could associate to each pattern. Consequently, the symbol patterns are closely related to what we freely call “information”. Being exposed to a message, our brain needs to find some patterns—order—to interpret the information in a useful manner. The more the order we find in the patterns of the message, the more we can rely on what we think we are perceiving. Thus, order as perceived is a measure of information and therefore entropy is linked to information.

In 1948 Shannon [24] proposed a way to quantify symbolic entropy (see Eq (3)) to evaluate the amount of information required to transmit a message. Shannon’s expression operates over strings of known symbols. He applied his method to binary codes based on zeroes and ones. But we think the symbols do not have to be limited to single character symbols, i.e. a symbol could be a group of three zeroes and four ones (0001111). In that case the entropy should be computed considering each time the symbol ‘0001111’ appears as an accountable instance of that symbol. Also, there could be more than two elementary symbols in the alphabet used to code the message. Despite Shannon’s expression was originally intended to evaluate the cost of transmitting messages between devices, we use it in a ‘reverse’ mode: we strive to determine the ‘best’ way to group characters building up larger but cohesive symbols within a message with the objective of minimizing the cost of transmitting the message. We claim that after having a ‘best’ set of symbols, whose frequency distribution corresponds to the lowest (or nearly so) possible symbolic entropy value is a good representation of the structure of the language used for the message or description. We call this set the Fundamental Symbols, and the method used for its determination is the Fundamental Scale Algorithm [23]. The result is that the set of symbols *Y*_*i*_, which can reproduce the description with such a frequency distribution ***P***(*Y*_*i*_) that the entropy associated with, is minimal. The set grouping the Fundamental Symbols is regarded as the Fundamental Language B_*_. The asterisk as sub-index is used to recall that B_*_ is the result of an entropy minimization process. Thus, we can write

$$B_{*}=\;\left\{Y_{1},\ldots ,\;Y_{i},\;\ldots \;,\;Y_{D}\;,\;P\left(Y_{i}\right)\right\}\;.$$ (1)

In Expression (1), the diversity—the number of different symbols—is represented as *D*. Once we know the set of Fundamental Symbols along with the frequency of each Fundamental Symbol—equivalent to their probability distribution—we can compute its symbolic specific diversity and the entropy of each piece of music, applying Expressions (2) and (3). The specific diversity *d* is calculated as

$$d=\;\frac{D}{N},$$ (2)

where *D* is the diversity of language ***B***—the number of different symbols in the description—and *N* is the total number of symbols, repeated or not. A version of Shannon’s entropy *h* [24], generalized for languages composed of *D* symbols, is used to compute the quantity of information describing each music piece. The probabilities of occurrence of symbols *Y*_*i*_ are the components of the one-dimensional array ***P***:

$$h=-\;P\;log_{D}\;P.$$ (3)

The modeling of descriptions by means of their representation with its Fundamental Symbols is a powerful tool that reveals, at least partially, the structure underneath the description of the system. But the process to obtain these Fundamental Symbols is lengthy, and although it is solely based on the Minimum Description Length Principle, its implementation may look cumbersome and difficult to follow, causing a lack of confidence in the results it produces. To illustrate the representation of texts by means of the Fundamental Symbols, we analyzed a segment of a MIDI file and a short English text. The results are presented in Table 1. Our choice of an English text is due to the unintelligibility to the naked eye of any music file text. The reader can verify that there is no overlap in the instances of the symbols, while symbols leave no empty space in the original message.

**Table 1:** Fundamental symbols leading to minimal entropy computed for short texts. On the right, the object text is a tiny segment of the MIDI representation of the 4^th^ movement of Beethoven’s violin concerto. On the left, the object text is an English text written for the purpose of this example. Symbolic models of the original texts were obtained by applying the Fundamental Scale Algorithm to the text’s objects.

Fundamental symbols of two short texts													
English text								MIDI Music file segment					
This is a group of little sentences built to evaluate the functioning of the fundamental scale algorithm. These sentences will be read in several ways. A specific way of splitting symbols will lead to the set of symbols that minimize the entropy. The entropy is computed according to the frequency of appearance of each symbol								‡¢$˜BT È- ¸Íé‚ú˜B ‰$˜BT‚ú˜B ‰$˜CT‚ú˜C $˜BT‚ú˜B $˜@T‚ú˜@ $˜BT‚ú˜B $˜@T‚ú˜@ ‰$˜BT‚ú˜B ‰$˜BT‚ú˜B ‰$˜@T‚ú˜@ ƒ$˜@T‚ú˜@ ƒ$˜BT‚ú˜B ‰$˜@T‚ú˜@ ƒ$˜@T‚ú˜@ ƒ$˜BT‚ú˜B ‰$˜@T ¸ÍéL¸ÍáÁ ¸Í€x˜@ $¸Í9Á ¸Í8ÁÉ¸Í7Á ¸Í6ÁÉ¸Í5Á ¸Í4ÁÉ¸Í3Á ¸Í2L˜@TL¸Í1ÁÉ¸Í0t˜@ $¸Í/ÁÉ¸Í.Á ¸Í-ÁÉ¸Í¤Á ¸Í+ÁÉ¸Í*Á ¸ÍóÁÉ¸ÍíH˜BT ¸Í2 È0—ú˜B $˜CT—ú˜C $˜BT—ú˜B $˜CT—ú˜C $˜BT ¸Í2 ¸Í3@¸Í4@¸Í5@¸Í6@¸Í7é˜B ¸Í8@¸Í9@					
Entropy *h* = 0.7552, Diversity *D* = 55, Ø = space								Entr. *h* = 0.7482, Div. *D* = 50, Ø = space					
**Symbol**	**Frq**.	**Symbol**	**Frq**.	**Symb**.	**Frq**.	**Symb**.	**Frq**.	**Symb**.	**Frq**.	**Symb**.	**Frq**.	**Symb**.	**Frq**.
Ø	43	l	6	ys.Ø	1	f	1	˜	40	1ÁÉ¸Í0	1	‡	1
e	27	ad inØ	1	Øli	1	g	1	Ø	28	ÍóÁÉ¸Í	1	¢	1
t	15	ymbols	1	c	3	m	1	‚ú	13	¸Í2 ¸Í	1	È	1
lgorithm. Th	1	mputed	1	bui	1	b	1	$	22	é‚ú˜	1	‚	1
symbols will	1	group	1	pli	1	r	1	B	22	Øƒ	2	ú	1
ntropy	2	n	5	ing	1	w	1	¸Í	11	—ú	2	€	1
frequency of	1	evalu	1	aØ	1	A	1	T	19	C	3	x	1
s	11	uncti	1	o	2	y	1	@	18	Í9Á	1	¸	1
specific wa	1	veral	1	h	2	.	1	3@¸Í4@¸Í5@¸Í6	1	L¸Í	1	Á	1
of	5	ad to	1	es	1	i	1	¸Í6ÁÉ¸Í5Á ¸Í	1	2 È	1	2	1
a	9	minim	1	iz	1			.Á ¸Í-ÁÉ¸Í¤Á	1	-	1	L	1
th	4	symbo	1	Th	1			¸Í+ÁÉ¸Í*Á ¸	1	CT	1	/	1
ccording	1	onin	1	co	1			T—ú˜C	2	ƒ	2	í	1
appeara	1	fund	1	nc	1			8ÁÉ¸Í7Á	1	t˜	1	H	1
This i	1	will	1	ch	1			‰	7	ÁÉ	1	0	1
								éL¸ÍáÁ	1	7é	1	9	1
								4ÁÉ¸Í3	1	8@	1		

The small experiment containing an English text is the only one we can attempt to interpret by looking for some meaning in the symbols found. Note that, in general, the algorithm does not find words. It does find suffixes, prefixes, roots and other character sequences that effectively reduce the entropy. In other cases, the algorithm finds the space character “” (represented in Table 1 as ‘Ø’). In fact, whatever the meaning of the space character, it is the most frequent symbol in English text, indicating that the space is among the most important symbols in the written English codification system. However, in most cases, it is difficult to find explanations for why a sequence of characters was selected as a Fundamental Symbol. We must simply accept that the Fundamental Scale Algorithm found a specific character sequence to be an effective entropy reducer, and therefore, it ends up being a Fundamental Symbol of that particular description.

Tracking meaning for the symbols found in the MIDI text is even more difficult. The MIDI file text is the result of coding the superposition of many sonic effects such as pitch, volume, timbre and rhythm—and all those effects for several instruments playing at the same time. Yet, the set of Fundamental Symbols along with their frequencies constitute a model of a MIDI file capable of capturing the essence and even subtleties of the sound associated with a performance of a music piece. It is worth emphasizing that our purpose is to develop methods for information pattern detections with no knowledge of the rules of the language used to code that information. Using some knowledge of coding protocols (such as MIDI) goes against our own objective and the generality of the method.

### Symbol frequency profiles

The value of entropy *h* is a good basis for the comparison of descriptions. But it may not be enough to properly represent the many dimensional differences of entities such as those we are dealing with. For that reason, we complement our treatment of each music piece with the shape associated with the values of array ***P***. To obtain the shape, we ranked the symbols according to their appearance frequency ***P***(*Y*_*i*_) and plotted ***P***(*Y*_*i*_) vs Rank (*r*), both in logarithmic scales.

The frequency profile drawn with as many points as the symbol diversity represents a shape of *D* different dimensions associated with the musical piece. The profile shape can change in many different ways, thus having the capacity to represent many of the subtleties differentiating one musical piece from another.

The symbol frequency profile representing the complete set of fundamental symbols is made of as many symbols as symbolic diversity *D* indicates. The leftmost point in the profile represents the most frequently appearing symbol in the textual description. The one to its right is the second-most frequent symbol, and so on. This region of the profile is usually called the profile’s ‘head’. At the other extreme, at the right, the least frequent symbols are represented forming the so-called ‘tail’ of the profile.

## Scale downgrading

When comparing the shape of several frequency profiles, the different number of symbols for which each profile was created is a problem. To solve this, we present a method we called Scale Downgrading to represent a symbol frequency profile with a smaller number of symbols while keeping its general shape. The scale downgrading is a transformation applied over the original profile to determine which frequency of fictitious symbols—each one formed by joining profile-neighbor symbols—would have in order to maintain the general profile’s shape. It is important to highlight that the downgrading transformation eliminates whichever meaning any symbol may have had at the original scale. After a profile’s scale has been downgraded, each resulting symbol is the result of joining, totally or partially, several symbols. These symbols might be neighbors in the sense of their frequency but not in the sense of the places they occupy in the text. Therefore, the resulting downgraded scale symbols do not actually exist as a recognizable sequence of characters in the text, and for practical purposes, they do not have any physical meaning. They do, however, serve as a valuable way to normalize the descriptions of the shapes we use as one of the bases for comparison. Details of the mathematical formulation to compute the scale downgrading are shown in S1 Appendix.

### Distance to Zipf’s reference profile

Any symbol frequency profile built with the probabilities of encountering a symbol is a probability distribution. The symbols ordered according to their frequencies give these probability distributions the shape we are considering—hypothetically for now—characteristic of music style. After properly applying the scale downgrading transformation, the two distributions can be represented at the same scale, that is, both distributions with the same number of points. Under this condition, the difference between the two distributions can be evaluated as the Euclidian distance computed with each corresponding pair of probability points. Within this context, the Zipfian profile, whose shape is a straight line in the log-log graph, can be used as a common reference profile. Thus, comparing a symbol frequency profile with the Zipf reference profile provides us with a parameter to characterize music. We call this parameter the Zipf reference distance.

Euclidian distances *E* are computed to measure the distance between the shapes of two selected profiles. For a given downgraded diversity *S*, and a pair of profiles having frequencies *f*_*r*_ and *g*_*r*_ for the symbols occupying *r-*th place, the Euclidean distance *E* between the two profiles is computed as

$$E=\sqrt{\sum_{r=1}^{S}{\left(\text{log}f_{r}-\text{log}g_{r}\right)}^{2}}.$$ (4)

Note the use of the logarithms of the symbol frequencies to maintain the meaning of the shapes, since for these purposes, the graph axes are logarithmic. To compute the distance from profile *f*_*r*_ to the Zipf reference profile, we just need to replace gr with the Zipfian distribution shown in Eq (5)

$$g_{r}=\;\frac{g_{1}}{r},$$ (5)

where *g*_1_ represents the probability of the most frequent symbol.

### Higher order entropy

The use of the symbolic diversity *d* and the symbolic entropy *h* to characterize music pieces allowed for differentiating among genres of music. However, two pieces of music, even though having different ranked frequency profiles, may share similar values of entropy. When this is the case, the difference between two profiles can be described as the way they ‘oscillate’ around their respective middle line. Looking for ways to evaluate the effects of these oscillations, we explored the value of the entropy associated with these oscillations. Therefore, we elevated the comparison of descriptions to a finer level of detail. Details of the mathematical formulation to compute the Higher Order Entropy are shown in S2 Appendix. In this work, we refer to the 2^nd^ order entropy with the symbol *h*, adding a superscript between brackets (*h*^*[2]*^).

### Music selection

Music is the result of the superposition of a vast variety of sounds. But music sounds reproduce not only to the information written on the music sheet but also to the addition of small differences introduced by the interpreter. Music is thus the result of a large number of different symbols to form sound sequences. These sound sequences are included in the file produced by the recording of a musical piece. Despite the unreadable condition of any of these files for humans, the files contain all the information regarding the music, and thus, we can appreciate this information as music when we reproduce the file and hear it. Due to the limitations of the Fundamental Scale [23] Algorithm and the enormous complexity of most conventional music recording formats, we had to rely on MIDI file coding to discretize the symbols forming these pieces of music while keeping the computations within a feasible condition for our algorithm in its current condition. Using formal music recording formats such as.MP3,.MP4 or.WAV is still desirable and a matter of further improvement of technical aspects of the Fundamental Scale Algorithm [23]. Nevertheless, MIDI music provides the conditions for us to advance with this study.

Table 2 shows a synthesis of the music selection we used as a subject to apply the entropy measurement method. The selection includes pieces from Classical and popular music of different genres. Our music library is organized as a tree. To have some reference of the place where a music piece, or group of pieces, is located within the tree, we assigned a name to each tree level. Table 2 also shows this classification structure fed with more than 450 pieces from 71 composers and 15 different periods or types of music.

**Table 2:** Music classification tree *MusicNet* and the data associated with top levels of the tree.

MusicNet												
							Specific diversity		Entropy		2^nd^ order entropy	
	Type	Period Style		Genre	Comps.	Pieces	Ave.	Std. Dev.	Ave.	Std. Dev.	Ave.	Std. Dev.
West.	Academic	Medieval			12	41	0.062	0.026	0.649	0.048	0.949	0.037
		Renaissance			10	31	0.048	0.016	0.622	0.037	0.935	0.041
		Baroque			8	55	0.039	0.013	0.581	0.057	0.911	0.050
		Classical			7	46	0.039	0.019	0.567	0.059	0.895	0.049
		Romantic			13	89	0.049	0.021	0.602	0.068	0.914	0.061
		Impressionistic			4	34	0.050	0.015	0.582	0.052	0.921	0.044
		20th Century			8	35	0.052	0.017	0.559	0.057	0.888	0.062
	Popular / Contemp.			Movie Themes		18	0.048	0.010	0.615	0.051	0.934	0.033
				Rock	5	24	0.041	0.010	0.585	0.043	0.919	0.045
			Venezuelan	Traditional	>20	56	0.049	0.014	0.540	0.056	0.929	0.036
Asian	Traditional		Hindu-Raga	Raga	Several	14	0.083	0.019	0.697	0.061	0.974	0.026
			Chinese		Several	12	0.048	0.015	0.582	0.038	0.915	0.046

## Results

All pieces of music were organized into a classification tree we refer to as *MusicNet*. By computing the Fundamental Scale to all leaves of *MusicNet*, we were able to obtain the fundamental symbols of each music piece included in our dataset as well as for each music subset defined by composer, type, genre, period, or any other characteristic property of the included music. *MusicNet* is too lush to be extensively presented here. But we include the upper levels of the tree in Table 2 and a link that allows access to the whole tree in S1 DataLink. Table 2 displays the datasets of MIDI music used for our tests and values of specific diversity, entropy and 2^nd^ order entropy accompanied with their respective standard deviations.

### Diversity and entropy

Diversity and entropy are quantitative characterizations of communication systems. Within the scope of a communication system, the diversity and the entropy may reveal differences regarding style or even period of its evolution. All pieces of our music library are organized into three groups: occidental academic, traditional and Rock/Movie Themes.

**Fig 1:**
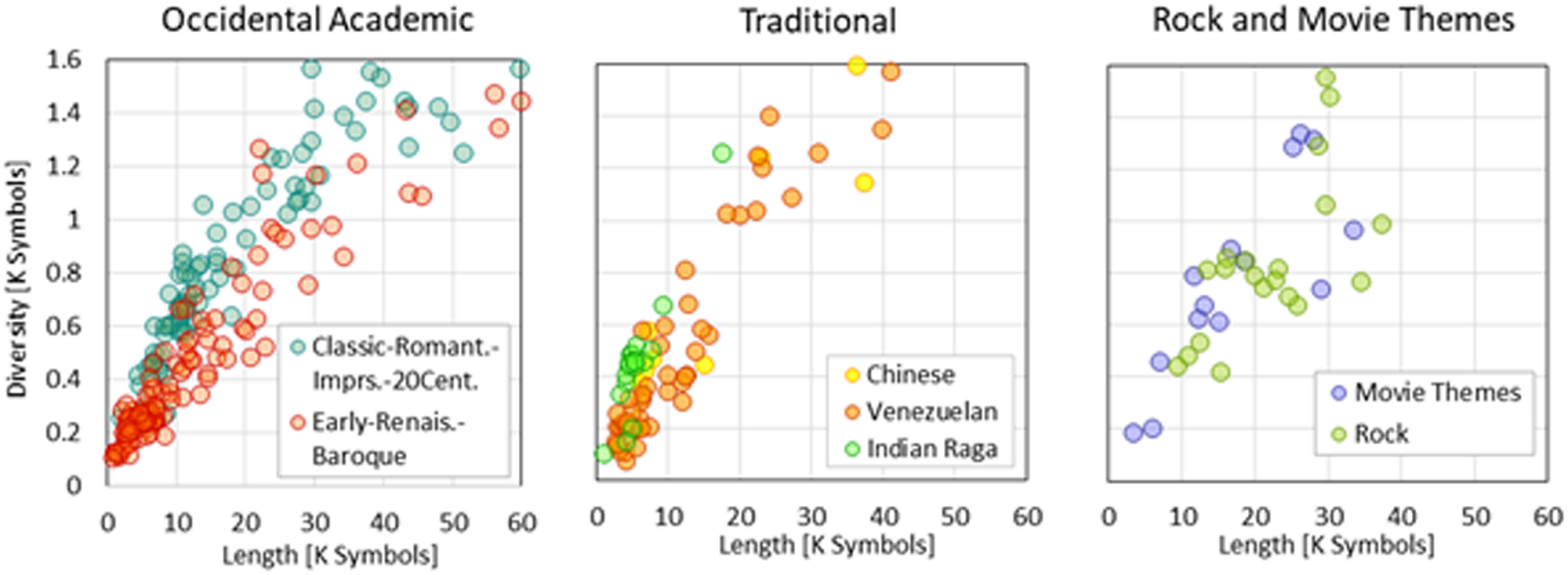
Diversity as a function of piece length measured in symbols for different classes of music. Each bubble represents a piece of music. The vertical axis represents the symbolic diversity *D* expressed in thousands of symbols. The horizontal axis represents the length *L* of the piece description expressed in symbols.

**Fig 2:**
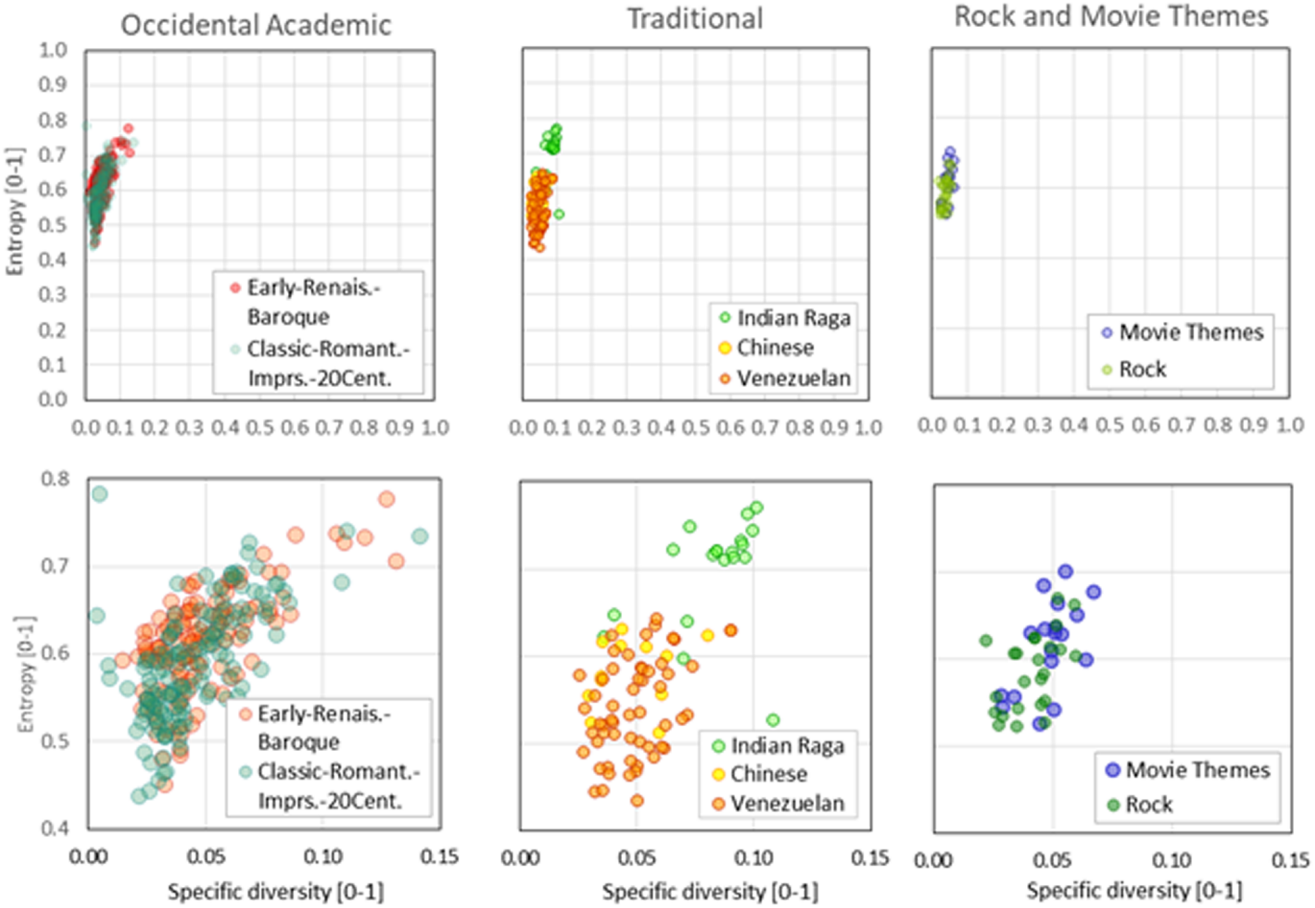
Entropy as a function of specific diversity for different classes of music. Each bubble represents a piece of music. The vertical axis represents the entropy *h*. The horizontal axis represents the specific diversity *d = D/L*. Top and bottom row show the same graphs presented at different scales.

Figs 1 and 2 are included to visually show how different styles and genres produce clustering of the values of some properties. Fig 1 shows that several music groups express with several symbolic diversities. According to Heap’s Law, for any description, the number of different symbols is expected to increase as the description’s length grows. The rates at which these growths occur—the slopes of the clouds of dots—, are evidence of different behavior among groups being compared. Fig 2, represents the same classes of musical pieces, grouping around different sectors of the entropy-specific diversity plane, implying a sensible distinction in the representative distribution of symbol frequency (entropy) for the music groups as selected.

### Information profiles

**Fig 3:**
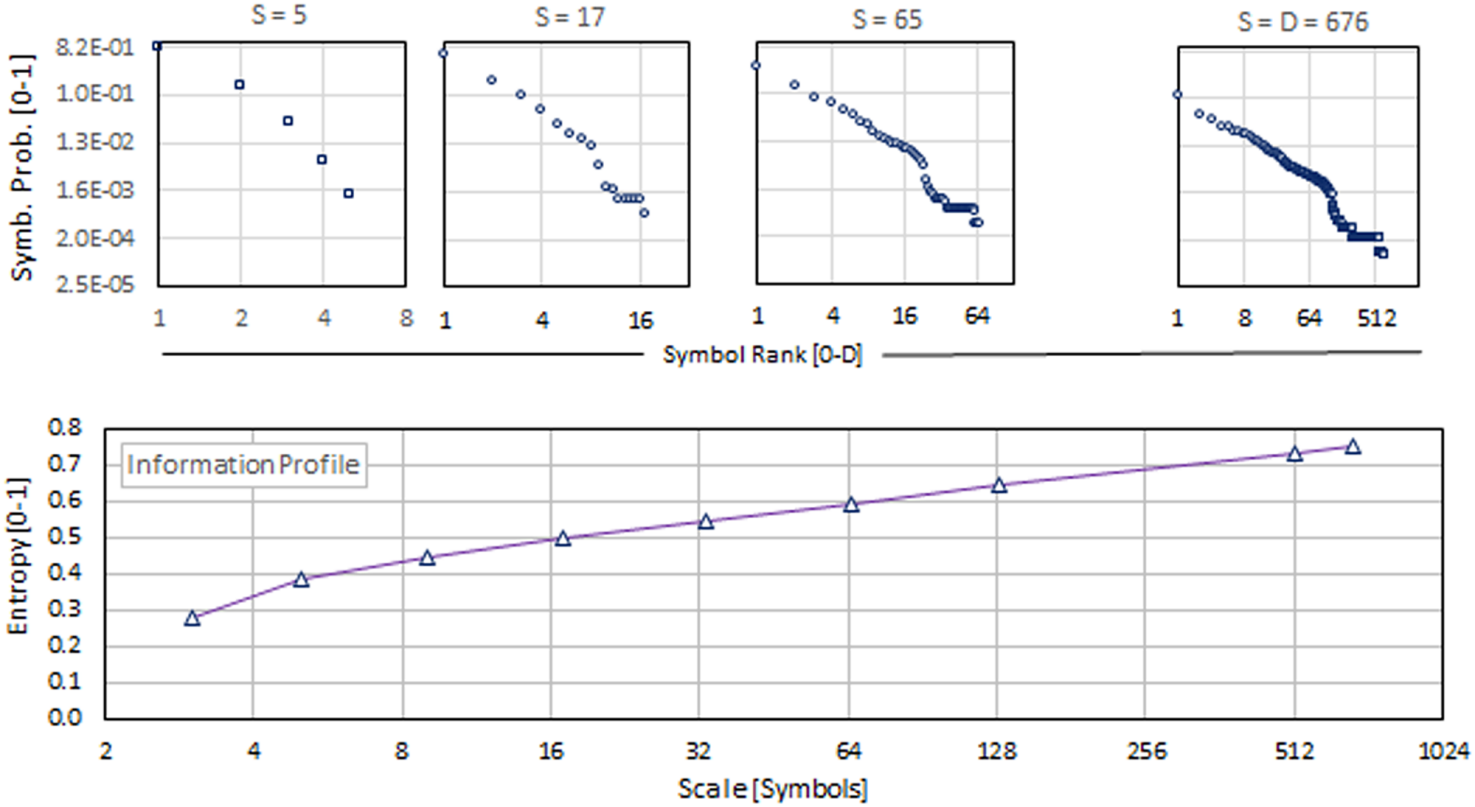
Variation of frequency profiles for several degraded scales and information profiles calculated for *Hindu-Raga*.*Miyan ki Malhar*.

We are interested in knowing the effect of degrading the scale of observation of musical descriptions. Prior to this computation, we know that degrading the scale—equivalent to viewing the system from a remoter perspective—means observing fewer details. Therefore, as the number of symbols used in the description decreases, we expect to obtain less information. Thus, there are at least two reasons to inspect these information profiles: (a) to evaluate whether they capture information about the music’s type or class and (b) to obtain a sense of the minimal degraded diversity that maintains the essence of the system by showing a shape that resembles the description at its original symbol diversity. The use of this *minimal degraded diversity* allowed us to compare the shapes of many music frequency profiles at the same diversity, a condition needed for a fair comparison. The downgraded values of the diversity were selected so that at any scale, the number of degrees of freedom of the symbol frequency profile (symbolic probability profile) is a power of *2*. The number of degrees of freedom of any probability distribution is *k*– 1, *k* being the number of different categories in the distribution. Thus, the number of different symbols considered for each degraded symbol diversity is *S* = 2^*i*^ + 1, and *i* is a positive integer. Examples of degraded symbol frequency profiles are shown in Fig 3 and Figures A and B in S1 Fig. To obtain them, we started from the description at their original symbol diversity *D* and degraded the observation scale *S* by applying Equations C, D, F and G in S1 Appendix. Amount of information (the entropy) values were computed for each of these profiles, thus providing the data needed to build the information profiles for several pieces of music. These information profiles are presented at the bottom of Fig 3 and Figures A and B in S1 Fig.

The information profiles show the scale of observation in the vertical axis and the scale of observation—the diversity *S* of symbols used in the description—in its horizontal axis. These graphs have been called by two different names: researchers who consider Shannon’s information [24] as a direct measure of complexity [25,26] call them *Complexity Profiles*; those who consider complexity as the pseudo-equilibrium [27–30] the system reaches when it bounds its disorder by self-organizing its symbols prefer to call these graphs *Information Profiles*. These names, which refer to the same type of graph, arise from the different interpretation of complexity. The first group of researchers see complexity as proportional to the length of the symbolic description, while the latter group pays more attention to the system’s activity to keep itself organized. Although these names refer to different concepts, both seem to be valid. In any case, these information profiles show how sensitive are the description lengths of a MIDI piece to the change in the observation scale, represented here by the downgraded diversity.

When comparing the information profiles for the *Hindu-Raga*.*Miyan ki Malhar* with the other two music pieces included in S4 Fig, it is visually clear that the Hindu-Raga piece is differentiated by showing a promontory in the profile at a diversity *S* = 17 that none of the other present at that scale. But the downgraded diversity *S* = 17 is not detailed enough to recognize the slight differences between the profiles of *Beethoven*.*Symph9*.*Mov_3* and *LAURO*.*Antonio-ValsVenezolanoNro3*.*Natalia* included in S4 Fig. To find visually different profile shapes among the three samples analyzed, we had to inspect the profiles with a diversity *S = 129*. With that level of refinement in the profile drawing, we were able to distinguish each music piece’s profile from another. We thus selected this diversity value (*S* = 129) as the diversity we should downgrade all pieces to in order to obtain characteristic property values for each piece.

### Symbol frequency profiles

**Fig 4:**
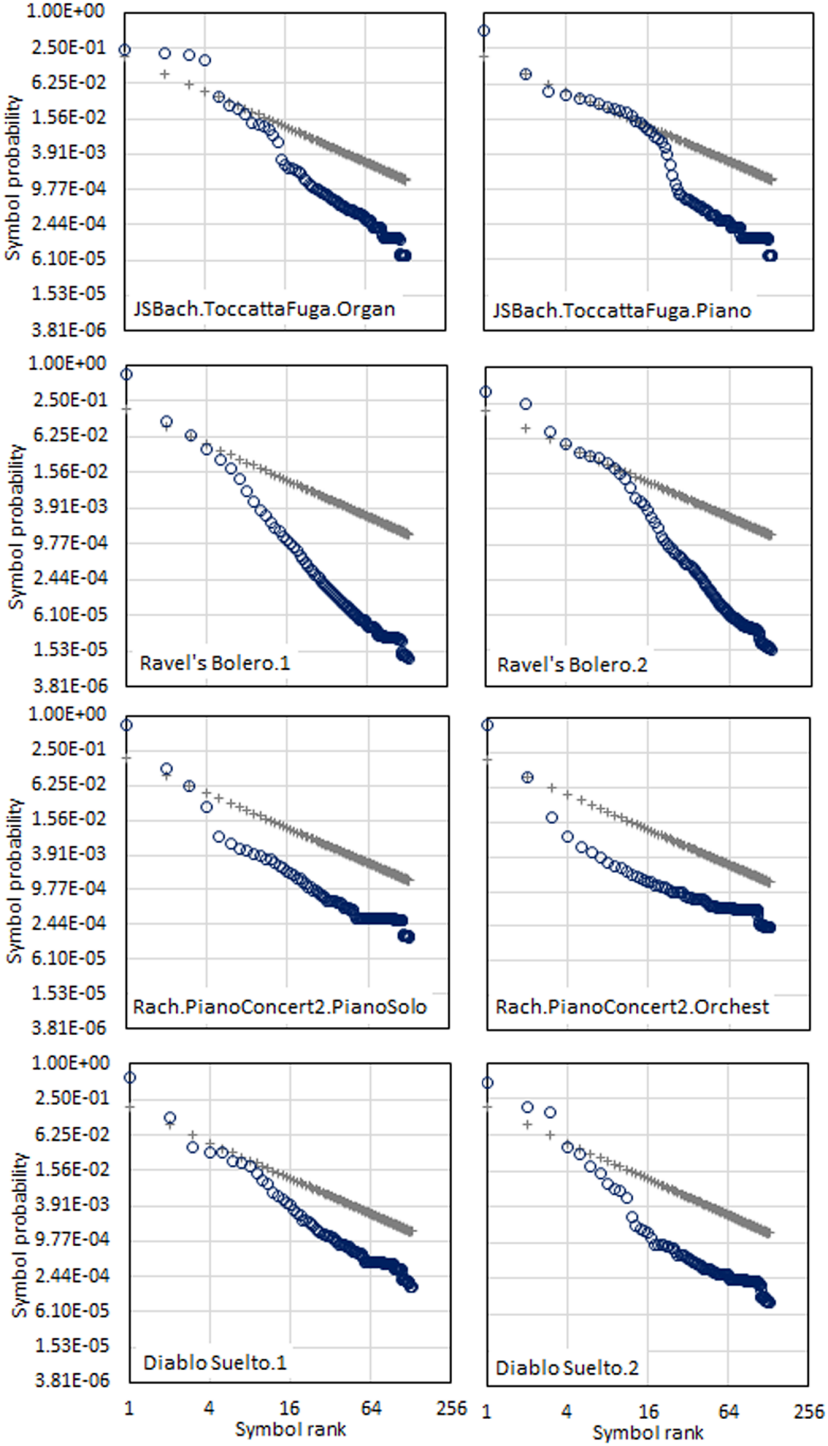
Symbol-ranked frequency profiles for eight performances grouped by pairs associated with the same music piece. Pairs of graphs show two performances of Bach’s Toccata & Fugue, Ravel’s Bolero, Rachmaninov’s Piano Concerto #2 and the Venezuelan waltz ‘El Diablo Suelto’. Each circle represents a symbol. As a helpful reference, each graph shows a Zipf profile represented by grey ‘+’ signs. The vertical axis is the probability of encountering a symbol with the text. The horizontal axis shows the rank of the symbols according to their frequency. Both axis scales are logarithmic. Links to sound: JSBach.ToccataFuga.Organ, JSBach.ToccataFuga.Piano, Ravel's Bolero.1, Ravel's Bolero.2, Rach.PC2.PianoSolo.mid, Rach.PC2.PianoAndOrchestra.mid. DiabloSuelto1.mid, Diablo Suelto 2.mid.

The hypothetical association between the shape of the frequency profiles and the identity of each music piece was considered. To assess this hypothesis, we rely on two distinct interpretations of four pieces of music. After obtaining the fundamental symbol set for each piece of music, we downgraded the scales to 129 symbols. The resulting profiles are shown in Fig 4, along with Zipf’s reference profiles. The graphs suggest that the symbol frequency profiles built with the Fundamental Symbols capture some of the essence of music pieces and represent that essence by means of a profile’s shape. This is, however, a subjective statement that should be qualified, at least for the small set of pieces performed more than once in our sample of music.

Table 3 shows the results of computing the distance between pairs of pieces. For each pair of pieces, the distance reveals how distant the shape of a profile is from another. We expected this distance be minimum for those pairs formed with the two different performances of the same piece. In general, the method worked well, indicating a short distance separating different performances of the same piece. Only Diablo Suelto.Perf.2 appeared closer to Toccata and Fugue with organ, thus indicating that this method is not infallible.

**Table 3:** Distances between profile pairs taken from profiles shown in Fig 1. Pairs of interpretations of music pieces are compared against each other and with respect to the Zipf reference profile. The comparison consists of the computation of the Euclidian distance over the logarithmic difference for each dimension, as indicated in Eq (2).

Distances between profiles										
			**Toccata & Fugue**		**Ravel's Bolero**		**Rachmaninov PC2**		**Diablo Suelto**	
		**Zipf's Ref**.	**Piano**	**Organ**	**Perf. 1**	**Perf. 2**	**Piano Solo**	**Orchestra**	**Perf. 1**	**Perf. 2**
**Toccata & Fugue**	**Piano**	11.27	0	**2.24**	8.31	6.11	3.81	5.27	3.66	3.39
	**Organ**	10.96		0	8.34	6.32	3.03	4.50	3.11	**2.18**
**Ravel's Bolero**	**Perf. 1**	19.12			0	**3.09**	9.68	11.18	10.92	8.28
	**Perf. 2**	17.03				0	8.25	9.82	9.00	6.86
**Rachmaninov PC2**	**Piano Solo**	9.86					0	**1.94**	2.32	2.00
	**Orchestra**	8.81						0	2.59	3.43
**Diablo Suelto**	**Perf. 1**	8.27							0	3.15
	**Perf. 2**	11.16								0

The profile distances to the Zipf reference also signal closeness between performances of the same music piece. Again, only the most freely interpretable piece, ‘*El Diablo Suelto’*, breaks the tendency to place the performances of the same music piece as the closest. But whenever the music piece performance sticks to the original musical arrangement, as it must for academic music, the profiles’ shapes remain remarkably similar, and the distances to Zipf’s reference are very close in magnitude.

We also propose the use of these profiles as a way to visualize the differences between classes of music. Each profile has *D* − 1 degrees of freedom. This means the profile’s shape can be altered in *D* − 1 different ways by modifying the frequency of the *D* different symbols that make up the description of the musical piece.

**Fig 5:**
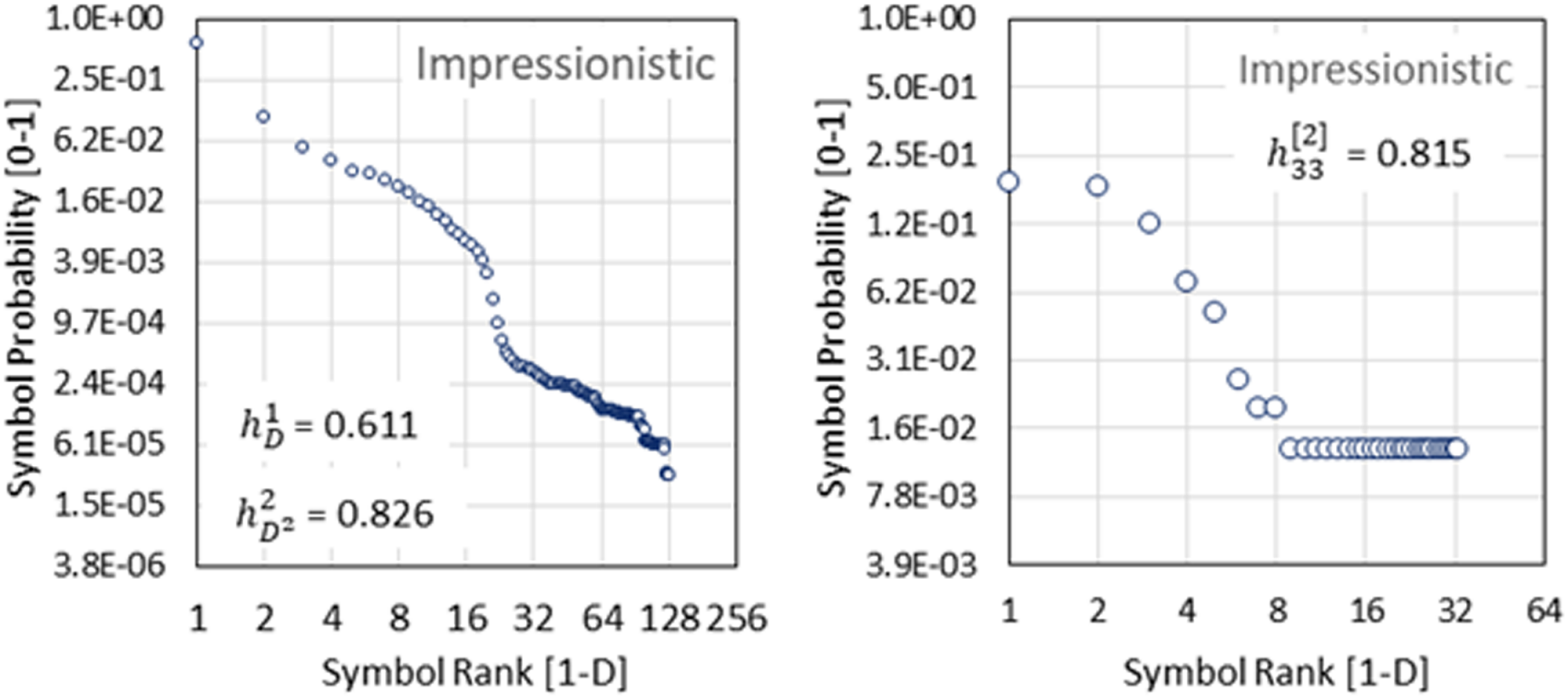
Symbol-ranked frequency profiles for Impressionistic music. Graph (a. Left) shows the traditional symbol profile. Graph (b. Right) shows the profile for the 2^nd^ order symbols.

An option to sense the profile’s shape around its middle straight line is to build a distribution based on the distance from the points representing each symbol's frequency to this straight line. Afterwards, a property we refer to as the Second Order Entropy can be computed. Details on the meaning of the Second Order Entropy are included in S2 Appendix. Fig 5 illustrates the symbol frequency profiles computed for our sample of Impressionistic music. Graphs (a) and (b) show first and second order symbol profiles correspondingly. Frequency profiles computed for all the groups of music contained in our data set are included in S2 and S3 Figs. All frequency profiles in S2 Fig were computed at a scale or downgraded diversity *D* = 129 using the numeric values of the probability of each symbol and each style of music, which are included in S1 Table. The 2^nd^ order frequency profiles shown in S3 Fig were all computed at a downgraded diversity *D* = 33.

When observing these frequency profiles, a reasonable question arises: Are these profiles capable of depicting the organized change that might be produced by an evolutionary process of music? The seven graphs corresponding to academic music, from Medieval to 20^th^ Century music, suggest that the answer is *yes*. For most periods and music styles, the frequency profiles exhibit two easily recognizable regions: a higher-ranked frequency region located toward the head of the ranked distribution and a second region at the right of the ranked distribution that extends to the symbol rank's cut-off value, where sometimes an elbow-shaped profile appears near the last ranked symbol at rank *D = 129*. For Medieval music, the distribution head's region occupies most of the profile range, showing a bow-shaped profile. While the academic type of music covers the time until the 20^th^ Century period, this bowed section progressively shortens until the transition of the two regions reaches the middle of the logarithmic horizontal axis. The last tail elbow also softens until it disappears at the Classical music profile. The slope at the transition zone also shows a gradual increase from the Medieval music, where the transition zone is very soft, up to the 20^th^ Century music, which shows a rather stiff transition zone. The vertical range of the profiles also grows as the time period progresses. With the sole exception of Impressionistic music, all other considered styles of academic music require a larger range of frequency values in the vertical axis when compared with the previous music period.

When looking at traditional and popular music, we observe a shorter vertical range of values if compared against the academic music profiles. From all non-academic music considered, Hindu-Raga music exhibits the flattest profile, while Chinese music has the steepest. The comparison of these profiles suggests that it is possible to capture structural music differences by observing these shapes. On the other hand, there are profile similitudes between some pairs of classes of music. Baroque music and Rock music, for example, have similarly shaped profiles. Also, the music from the Impressionistic and Chinese periods exhibits similar overall profiles. However, reducing the profile shapes to a quantifiable index proves difficult and perhaps overly simplistic. In this sense, the inclusion of an additional characteristic, like our recently defined 2^nd^ Order Entropy, seems justified.

### Recognizable music genres and styles

Using the entropy and the Zipf reference distance as parameters, musical pieces were characterized. We grouped all pieces of academic music by composer, and for traditional and popular music, by composer or style.

Classification of music genres and styles is usually guided by chronological and geographical conditions. Actual musical structure should represent the dominant musical aspects of music. However, music classification is usually dominated by circumstances related to its origin. Thus, currently accepted music classification schemes may not properly split musical groups in terms of the actual structure of each piece or group of pieces. Yet, because musical style is a field of enormous diversity, we must start from musical group categorizations and measure the actual differences among those groups.

Tables 4 and 5 show the entropy and the Zipf’s reference distance for the musical pieces’ groups included in this study. Mean values and standard deviations are the parameters used to compare these groups. Most groups are defined according to the corresponding composer. The composers’ names are not relevant at this point. For both Tables, the groups are organized according to the time period for academic music and by geographical condition for traditional and popular music. Averages and standard deviations for the pieces of each composer are also presented in Tables 4 and 5.

**Table 4:** Entropy and distance to Zipf’s reference for academic music grouped by composer and period.

Entropy and distance from Zipf's ref. of academic music grouped by composer														
	0.Medieval		1.Renaiss.		2.Baroque		3.Classical		4.Romantic		5.Impress.		6.Twentieth	
	Entr.	Zipf's dist.	Entr.	Zipf's dist.	Entr.	Zipf's dist.	Entr.	Zipf's dist.	Entr.	Zipf's dist.	Entr.	Zipf's dist.	Entr.	Zipf's dist.
**Ave**	0.658	0.126	0.643	0.127	0.624	0.129	0.598	0.209	0.614	0.219	0.634	0.221	0.578	0.239
**S.d**.	0.101	**0.041**	0.032	**0.035**	0.055	**0.026**	0.049	0.062	0.062	0.072	0.040	0.074	0.056	0.086
Data. Each number corresponds to a group of pieces by a composer:														
	0.643	0.133	0.593	0.174	0.526	0.163	0.593	0.273	0.516	0.351	0.591	0.289	0.609	0.193
	0.585	0.130	0.663	0.186	0.579	0.156	0.541	0.255	0.522	0.327	0.689	0.122	0.526	0.262
	0.618	0.140	0.625	0.128	0.692	0.083	0.548	0.251	0.640	0.218	0.657	0.178	0.556	0.384
	0.779	0.065	0.585	0.144	0.657	0.125	0.650	0.133	0.648	0.186	0.602	0.296	0.600	0.209
	0.612	0.116	0.679	0.090	0.649	0.130	0.657	0.133	0.522	0.298			0.606	0.133
	0.575	0.186	0.678	0.112	0.639	0.117			0.634	0.180			0.686	0.118
	0.706	0.065	0.652	0.100					0.690	0.146			0.502	0.337
	0.626	0.154	0.650	0.099					0.627	0.274			0.603	0.202
	0.660	0.145	0.630	0.156					0.658	0.135			0.510	0.311
	0.526	0.181	0.672	0.080					0.674	0.161				
	0.902	0.069							0.593	0.188				
									0.694	0.125				
									0.560	0.255				

The averages for entropy and Zipf’s distance for academic music are 0.543 and 0.181 respectively. The standard deviations for entropy and Zipf’s distance for academic music are 0.069 and 0.078 respectively.

**Table 5:** Entropy and distance to the Zipf’s reference for traditional and popular music grouped by composer and style.

Entropy and distance from Zipf's Ref of traditional and popular music grouped by composer												
	Chinese		HinduRaga		MovieThemes		Rock		Venezuelan		Venezuelan	
	Entr.	Zipf's dist.	Entr.	Zipf's dist.	Entr.	Zipf's dist.	Entr.	Zipf's dist.	Entr.	Zipf's dist.	Entr.	Zipf's dist.
**Ave**.	0.582	0.266	**0.696**	0.146	0.615	0.254	0.588	0.260	**0.534**	0.305		
**Std.Dev**.	**0.038**	0.057	0.064	0.087	**0.051**	0.095	**0.042**	0.046	0.062	0.122		
Data. Each number corresponds to a group of pieces:												
	0.5710	0.321	0.720	0.122	0.556	0.175	0.520	0.205	0.522	0.277	0.629	0.101
	0.5560	0.334	0.719	0.106	0.701	0.087	0.635	0.310	0.423	0.461	0.489	0.195
	0.6232	0.296	0.770	0.083	0.600	0.304	0.630	0.268	0.582	0.250	0.467	0.210
	0.6158	0.193	0.744	0.089	0.678	0.230	0.580	0.308	0.494	0.316	0.496	0.190
	0.5226	0.319	0.732	0.110	0.684	0.115	0.577	0.209	0.472	0.553	0.470	0.342
	0.6309	0.185	0.762	0.115	0.630	0.140			0.506	0.272	0.496	0.212
	0.5989	0.184	0.709	0.128	0.525	0.372			0.586	0.413	0.619	0.226
	0.5766	0.254	0.727	0.110	0.630	0.197			0.561	0.190	0.526	0.355
	0.5109	0.338	0.716	0.121	0.651	0.341			0.625	0.144	0.501	0.197
	0.5547	0.225	0.713	0.139	0.545	0.296			0.521	0.450	0.433	0.248
	0.6099	0.301	0.597	0.335	0.598	0.418			0.601	0.349	0.521	0.406
	0.6116	0.246	0.712	0.124	0.635	0.335			0.634	0.268	0.446	0.467
			0.640	0.398	0.636	0.211			0.573	0.230	0.643	0.149
			0.721	0.104	0.558	0.208			0.584	0.302	0.465	0.509
			0.748	0.091	0.541	0.346			0.501	0.401	0.623	0.344
			0.720	0.122	0.609	0.371			0.444	0.331	0.512	0.485
			0.505	0.291	0.664	0.167			0.543	0.404	0.554	0.199
			0.646	0.102	0.629	0.254			0.582	0.385	0.521	0.581
			0.622	0.095					0.528	0.345	0.605	0.131
									0.512	0.303	0.464	0.210
									0.629	0.101		

The averages for entropy and Zipf’s distance for traditional and popular music are 0.603 and 0.246 respectively. The standard deviations for entropy and Zipf’s distance for traditional and popular music are 0.076 and 0.100 respectively.

**Fig 6:**
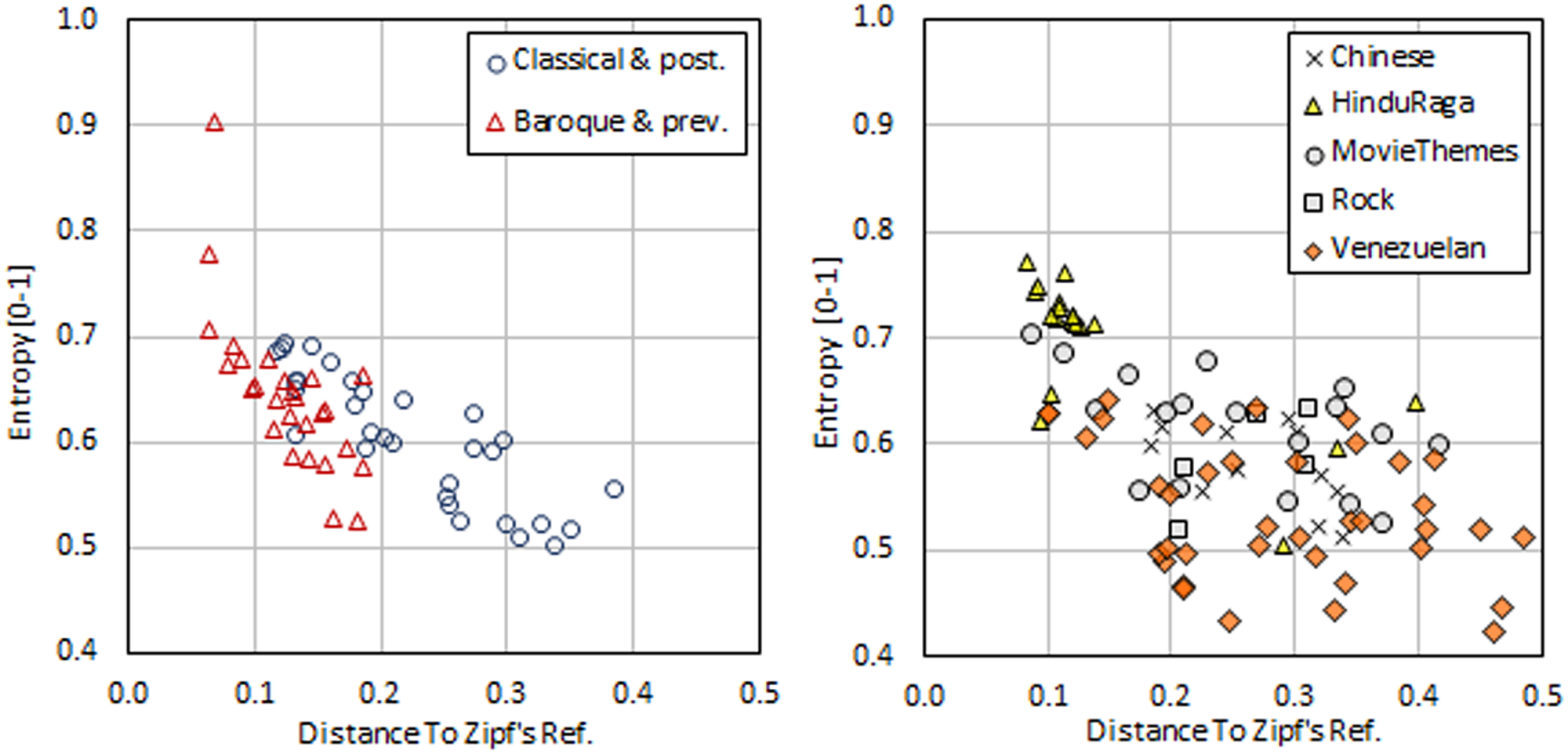
Entropy vs distance to Zipf’s reference for separable groups of music. Left: academic music. Right: traditional and popular music.

It is straightforward to see that for academic music, the baroque, renaissance and medieval periods exhibit the lowest Zipf’s reference-distance standard deviations. This suggests that all other periods of music—Classical, Romantic, Impressionistic and Twentieth—are not easily distinguished by means of entropy and Zipf’s reference distance as properties. Taking advantage of the fact these two groups of music are formed with successive periods, we can consider two sub-groups of academic music. The first sub-group contains Baroque and previous music, while the second sub-group contains music from the Classical period and afterward. Fig 6 (left) shows entropy vs Zipf’s reference distance for the composers classified within each of these groups, which is the data shown in Table 4.

By similar reasoning, in comparing the standard deviations of entropy and Zipf’s reference distance for the groups of music included in Table 5, we recognize three sub-groups of music: Hindu-Raga, Rock-MovieThemes-Chinese and Venezuelan. Fig 6 (right) shows the data included in Table 5 represented in the plane entropy vs Zipf’s reference distance.

Fig 6 illustrates how the entropy and Zipf’s ref. distance for the selected music sub-groups have probability distributions with different mean values. Student t-Tests were performed to evaluate the likelihood of two of these distributions having the same mean values. The results are shown in Tables 6 and 7. The p-values are very low, and therefore, we can affirm that these groups tend to occupy different places in the space entropy-Zipf’s ref. distance.

**Table 6:** Student t-tests for entropy and distance to Zipf’s reference for academic music sub-groups.

Academic music separable groups				
	Baroque and Prev.		Classical and Post.	
	*Entropy*	Zipf's dist.	*Entropy*	Zipf's dist.
**Average**	0.6445	0.1269	0.6034	0.2234
**Std.Dev**.	0.0730	0.0360	0.0590	0.0761
t-Tests p-values:				
Baroque and Pre vs. Classical and Post.			Entropy	0.01171
			Dist. Zipf Ref.	**1.03E-07**

**Table 7:** Student t-tests for entropy and distance to Zipf’s reference for traditional and popular music sub-groups.

Traditional and popular music separable groups						
	Hindu-Raga		Rock.MovieThemes.Chinese		Venezuelan	
	*Entropy*	Zipf's dist.	*Entropy*	Dist. Ref.	*Entropy*	Zipf's dist.
**Average**	0.6959	0.1465	0.5998	0.2589	0.5054	0.3261
**Std.Dev**.	0.0872	0.0872	0.0484	0.0780	0.05710	0.0815
t-Tests p-values:						
Rock.MovieThemes.Chinese vs Hindu Raga					Entropy	**7.11E-08**
					Dist. Zipf Ref.	**7.94E-06**
Venezuelan vs Hindu-Raga. Entropy					Entropy	**1.62E-05**
					Dist. Zipf Ref.	**2.87E-05**
Rock.MovieThemes.Chinese vs Venezuelan					Entropy	**1.73E-06**
					Dist. Zipf Ref.	0.03116

To complement the significance tests over these groups of music, we conducted ANOVA Univariate Tests of Significance. We kept the Academic and Traditional music categories. We obtained the results shown in Tables 8 and 9. These results confirm significant values for the ratio *F* of the Zipf’s ref. distance variance and the variance of the same property for the whole subgroup. For academic music, this ratio ranges within values comparable to those for traditional and popular music (5.55 and 7.6, respectively), indicating that the distance to Zipf’s reference has approximately the same significance for the music styles contained in both groups. Considering entropy, the ratio of variances F indicates a much more significant value for traditional music (25.76) than for academic music (1.46). Actually, the results indicate that entropy by itself, does not recognize differences among the styles included for academic music.

**Table 8:** Univariate ANOVA tests for entropy and distance to Zipf’s reference for academic music sub-groups. Degrees of freedom = Number of groups– 1, *SS* = Sum of squares of variance errors, *MS* = Mean of squares of variance errors, *F = MS /* Degrees of freedom.

Results for Univariate ANOVA Test of Significance for Academic music groups									
		Entropy				Distance to Zipf's Reference			
	Degrees of freedomm	*SS*	*MS*	*F*	*p- value*	*SS*	*MS*	*F*	*p-value*
Intercept	1	18.98709	18.98709	4107.39	0.000000	1.619136	1.619136	391.32	0.000000
Style	6	0.04038	0.00673	1.456	0.212138	0.137689	0.022948	5.5463	0.000175
Error	51	0.23576	0.00462			0.211018	0.004138		
Total	57	0.27614				0.348707			

**Table 9:** Univariate ANOVA tests for entropy and distance to Zipf’s reference for traditional and popular music sub-groups. Degrees of freedom = Number of groups– 1, *SS* = Sum of squares of variance errors, *MS* = Mean of squares of variance errors, *F = MS /* Degrees of freedom.

Results for Univariate ANOVA Test of Significance for Traditional and Popular music groups									
		Entropy				Distance to Zipf's Reference			
	Degrees of freedomm	*SS*	*MS*	*F*	*p- value*	*SS*	*MS*	*F*	*p-value*
Intercept	1	21.86110	21.86110	6380.30	0.00000	3.646193	3.64619	338.4282	0.00000
Style	4	0.35305	0.08826	25.76	0.00000	0.327639	0.08191	7.6026	0.00003
Error	90	0.30837	0.00343			0.969651	0.01077		
Total	94	0.66142				1.297291			

We also performed ANOVA Multivariate Tests of Significance. The results, presented in Table 10, reveal consistency with previously presented tests. Interpreting the Wilks’ Λ values as suggested in [31] indicates that for the groups of academic and popular music considered here, the location of each group’s entropy and Zipf’s reference distance mean values explain approximately 50% of the music type they are associated with.

**Table 10:** Multivariate ANOVA tests for entropy and distance to Zipf’s reference for traditional and popular music sub-groups. Λ value = Wilks’ lambda value. *F =* Mean of Squared Errors */* Degrees of freedom.

Results for Multivariate ANOVA Tests of Significance											
		Academic music					Traditional and Popular music				
	Test	*Value*	*F*	*Effect df*	*Error df*	*p-value*	*Value*	*F*	*Effect df*	*Error df*	*p-value*
Intercept	Wilks	0.00358	6959.4	2	50	0.0000	0.00850	5189.7	2	89	0.0000
Style	Wilks	0.50079	3.442	12	100	0.0003	0.45872	10.602	8	178	0.0000

### Clusters and tendencies

**Fig 7:**
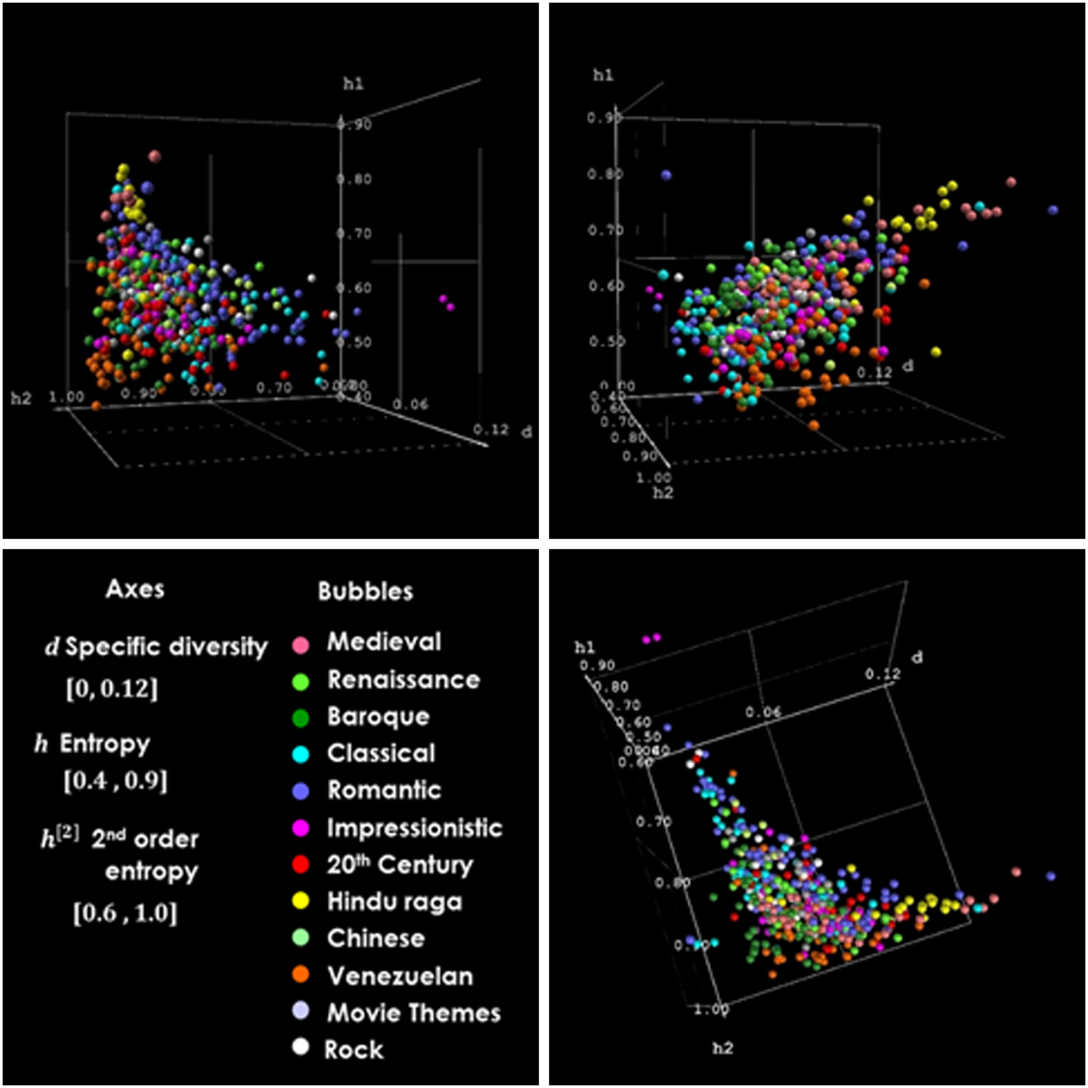
Three views of the representation of selected MIDI pieces in the space specific diversity, entropy, and 2^nd^ order entropy (*d*, *h*^*[1]*^, *h*^*[2]*^). Each bubble represents a MIDI piece.

The frequency profiles built lead yield values of symbolic diversity, entropy and 2nd order entropy for our selected set of MIDI-musical pieces. These values are available in full extension in S1 DataLink [32]. Fig 7 presents 3D graphs for the diversity *d*, entropy *h*^[1]^ and 2nd order entropy *h*^[2]^ of our music data sample.

In the graphs of Fig 7, each bubble corresponds to a single music piece. When a musical work is complex and can be divided by parts, such as suites, concerts and symphonies, each part is considered a single piece and is represented by a bubble. Fig 6 and S6 and S7 Figs show the average values of the same properties, but this time, they are computed for sets of musical pieces grouped according to music types and composer. Thus, in these figures, each bubble corresponds to a different music period/style or a composer. Three views of the same 3D plot are presented.

The graphs shown in Fig 7 may appear, at first glance, as a disorganized mix of bubbles representing music styles in our 3D space. Certainly, there are clusters of types of music sharing the local space. Therefore, it would be difficult to split some clusters according to their location. However, despite the difficulty of seeing through this dense cloud of bubbles, for some specific types of music, the separation of their cluster’s locations seems a feasible task. Medieval music (old-rose bubbles), for example, occupies a subspace of relatively high entropy and high diversity compared to the location of Renaissance music (light green bubbles). Following the chronological time direction, Baroque music (dark green bubbles) maintains the general tendency towards a reduction of its symbolic diversity *d* and its entropy *h* (represented as *h1* in the 3D graphs). Comparing Classical music (light blue) with Baroque, its predecessor in time, we observe a stabilization of diversity *d* and entropy *h* values; however, a noticeable reduction appears in the values of the second order entropy *h*^[2]^ (represented as *h2* in the 3D graphs).

**Fig 8:**
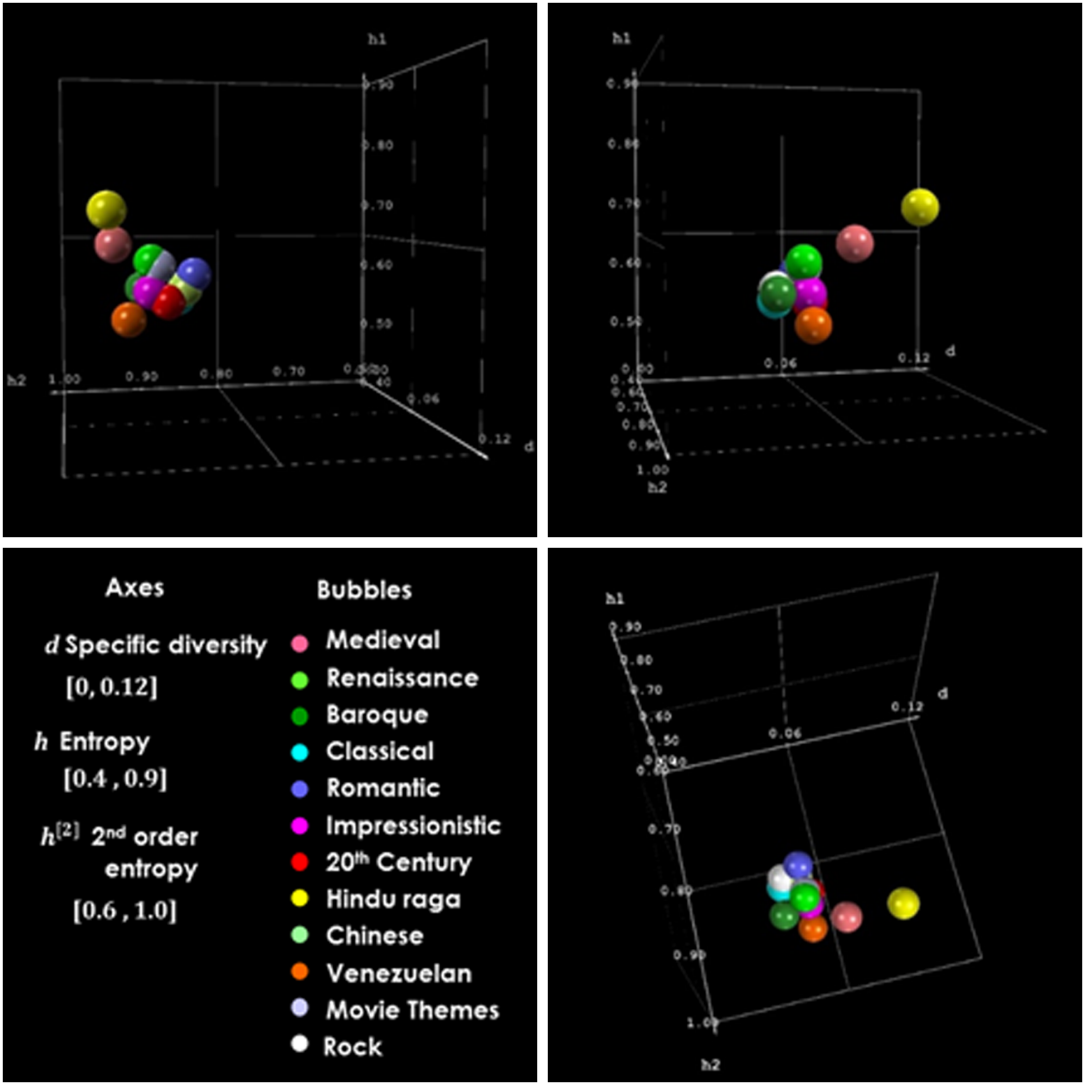
Three views of the representation of music period/style groups in the space specific diversity, entropy, 2^nd^ order entropy (*d*, *h*^*[1]*^, *h*^*[2]*^). Each bubble represents a group of music pieces sharing the same style/period.

On the other hand, if we consider ‘distant’ types of music, such as Hindu-Raga (yellow bubbles) and Venezuelan music (orange bubbles), there is very little or no overlap between the spaces where the bubbles are; these clusters occupy different spaces, and our representation allows them to be separated. Fig 8 reveals how all periods of academic music are located in different sectors of the 3D space formed by diversity *d*, entropy *h* and 2^nd^ order entropy *h*^[2]^. It is worth mentioning that in Fig 8, the size of the bubbles does not represent the dispersion of the music pieces grouped under a music style or period; thus, there is more overlap among types of music than suggested by the representation of the bubbles in the graph.

**Fig 9:**
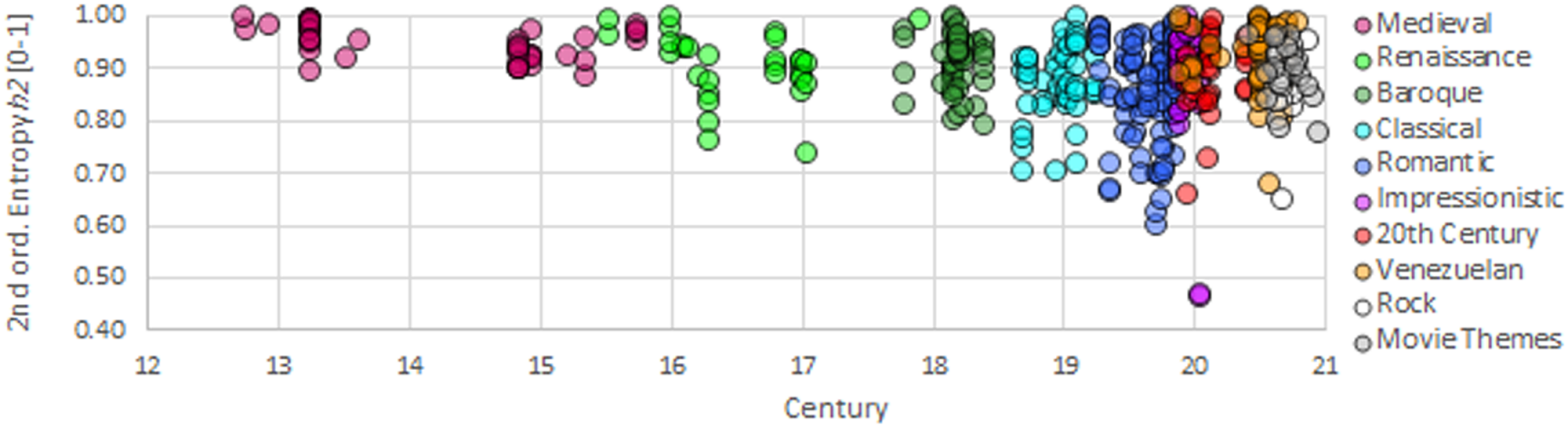
Change of the second order entropy over the last few centuries for genres and styles of music.

To have a quantitative sense of the overlap occurring for these clusters, we present the averages and standard deviations of the properties that characterize each type of music in our sample. Tables 11 and 12 show the results. To appreciate any tendency of specific diversity *d* and entropies *h* and *h*^[2]^ over time, we plotted these variables as functions of time. The resulting graphs are included in Fig 9 and S5 Fig. For the Chinese and Hindu-Raga music pieces, we do not have information about the time when they were composed. Therefore, we did not include these types of music in these graphs.

**Table 11:** Properties of western academic music.

Properties of western academic music								
		Medieval	Renaissance	Baroque	Classical	Romantic	Impress.	20th Century
	Num.Elem.	*41*	*31*	*55*	*89*	*46*	*34*	*35*
Specific diversity *d*	Average	0.0618	0.0479	0.0388	0.0390	0.0485	0.0500	0.0518
	Std.Dev.	0.0258	0.0159	0.0127	0.0192	0.0210	0.0150	0.0168
Entropy *h*	Average	0.6489	0.6219	0.5806	0.5662	0.6023	0.5819	0.5592
	Std.Dev.	0.0475	0.0373	0.0566	0.0585	0.0676	0.0521	0.0570
2nd order entropy *h[2]*	Average	0.9446	0.9014	0.9085	0.8668	0.8521	0.8829	0.8917
	Std.Dev.	0.0320	0.0629	0.0499	0.0693	0.0945	0.1153	0.0679

**Table 12:** Properties of some traditional and popular music.

Properties of popular and traditional music						
		Hindu Raga	Chinese	Venezuelan	Movie Thms.	Rock
	Num.Elem.	*14*	*12*	*56*	*18*	*24*
Specific diversity *d*	Average	0.0828	0.0476	0.0493	0.0485	0.0415
	Std.Dev.	0.0189	0.0153	0.0143	0.0104	0.0103
Entropy *h*	Average	0.6971	0.5818	0.5398	0.6150	0.5853
	Std.Dev.	0.0607	0.0380	0.0558	0.0511	0.0431
2nd order entropy *h[2]*	Average	0.9539	0.8608	0.9259	0.8915	0.8594
	Std.Dev.	0.0288	0.0777	0.0614	0.0104	0.0696

## Discussion

Music can be transmitted by sounds and by writing. However, the communication of music by writing lacks music’s essence and does not produce, at least not for most people, the emotions and sensations associated with a pattern of sounds. Music writing should be considered a useful tool for composing, making arrangements, recording, and teaching music. Thus, transferring musical information is possible by means of music sheets or other kinds of written music representation. However, we think that, rigorously speaking, written forms of music convey information instead of real music. Thus, we devote our discussion to some of the properties of the information associated with a set of MIDI files. Our purpose is to demonstrate that the properties of these texts, even though indirectly, can be used to characterize and, to some degree, depict the actual pattern of sounds we call music.

### Diversity and entropy

The dependence of Diversity *D* vs Length *N* is nearly linear. Only for short music pieces does the Diversity-Length curve show slight concavity. For all other ranges, the Diversity *D* of music can be modeled as a linear relationship with the length *N* of the music description. The slope change observed near the origin may be due to the English and Spanish overhead texts, which are generally included to start and end the MIDI files. These natural language segments are considered noise, and its presence should not have an important effect on the overall music description when the music piece is reasonably large in terms of symbols. Nevertheless, the specific diversity *d*, represented by slope *D*/*N*, keeps close to a constant value for every type of music, becoming a characteristic value that may distinguish one type or style of music from another. Fig 1 illustrates how the point clusters for different types of music tend to group around different lines, leading to different averages of specific diversity *d*, as shown in Tables 4 and 5. The specific diversity value measured for individual pieces ranges from 0.0183 (*Academic*: *Impressionistic*: *RAVEL*.*Maurice*, *Bolero2*) to 0.1341 (*Academic*: *Romantic*: *SAINTSAENS*.*Camille*: *CarnavalDesAnimaux*: *08*.*PersonnagesLonguesOreilles*). A complete set of values can be found in the link signaled in S1 DataLink.

As can be seen in Fig 2, the graphs show that entropy is aligned with a very stiff slope in the space entropy *h* vs specific diversity *d*. And even though the entropy values represented fill a wide range of values (from *0*.*45* to *0*.*8*), they seem to closely follow an average curve of the form *h* = *d*^*α*^, similar to those found for natural human languages in a previous work [33]. The large dispersion of entropy is, then, a consequence of the small range of specific diversities *d* where music is established. Nevertheless, the standard deviations of entropy observed in Tables 4 and 5 are generally small compared to the range of entropy averages, suggesting that entropy values capture some of the essence of the type or period of music and therefore justify its inclusion in a music entropy model. Values of the *2*^*nd*^ order entropy average range from *0*.*89* (*Academic*: *20*^*th*^ *Century)* to *0*.*97 (Asian*: *Traditional*: *Hindu-Raga*). The standard deviation is approximately *0*.*05* and is generally smaller than the range of variation of the average 2^nd^ order entropy from one group to another.

### Symbol frequency profiles

The frequency profiles shown in Fig 4 provide matter for interesting observations. By looking at the profiles of different performances of the same musical piece, we addressed the capability of the method to identify a musical piece. The first three pairs of profiles correspond to academic music pieces, while the last corresponds to a popular-traditional piece. Despite the little freedom academic music leaves to the performer to depart from the music sheet, these two versions of Bach’s Toccata & Fugue, Ravel’s Bolero and Rachmaninov’s Piano Concert exhibit recognizably different sounds. The performance of Ravel’s Bolero 2 offers neater sounds and the instruments are better defined, while performance 1 has excellent volume control along the whole interpretation. The two versions of Johan Sebastian Bach’s Toccata & Fugue and Rachmaninov’s Piano Concerto #2 are played with different instrumentations. Thus, the differences in sound are basically established by the characteristic timbre of the instruments and the persistence of the sounds. This last effect is very noticeable when comparing the organ version of Toccata & Fugue with its counterpart version with the piano. Despite the differences among the music pieces presented, the profiles show an undeniable correspondence.

At the bottom of Fig 1, we compare the profiles of two interpretations of ‘El Diablo Suelto’, a relatively well known traditional Venezuelan waltz that is typically used by interpreters to introduce important variations in the arrangement and while performing. These two performances are very different. The velocity, pitch, syncopation and ornaments used in each one make these profiles representative of noticeably different expressions of the same music piece. Yet, even with the important distance between these two interpretations, the similarities of the two profiles are visually evident, especially in the tails, thus strongly suggesting that the frequency profile may serve as a useful tool for identifying versions of the same musical piece.

Changing the focus from specific musical pieces and their identity to groups of music genres and styles and their characteristics, we proceeded with several comparisons. First, we inspect the shapes of the ordered frequency profiles for all types of music included in this study. Comparing them visually, we find similarities among the profiles of different types of music. Baroque and Rock show very similarly shaped profiles, as do the chronologically successive periods of the Romantic and Impressionistic. In another comparison, we find that Hindu-Raga and Venezuelan music have the flattest and steepest profile shapes, respectively, locating their shapes at opposite extremes of a scale somehow built to evaluate these shapes. The results presented in Tables 4–10 verify the significance of these qualitative observations of differences and similitudes for the profiles representing groups of music styles.

Tables 4 and 5 indicate that by using entropy and Zipf’s reference distance as characterizations of the frequency profiles, groups of music genres and styles can be formed to obtain almost separable distributions, thus making these music groups recognizable. Tables 6–10 show that parameter distributions for originally set groups of music are different, even though they present important overlaps, especially for those groups corresponding to close conditions, such as the chronological.

Finally, having statistically established the degree of significance of this method for the description of music styles and genres, a set of these 128 degree-of-freedom ‘fingerprints’ for groups of MIDI music is included in S6 Fig.

### Clustering of music genres and styles

By building larger sets of music, we have shown that musical genre and style define where, in the plane entropy vs Zipf’s reference distance, a piece of music is represented. The low p-values in Tables 6 and 7 show that the mean values of these parameters for the corresponding music sub-groups are definitively different. The graphs in Fig 6 also indicate that there is little overlap between these groups. For academic music in particular, the clouds of dots look almost separable. This is perhaps a consequence of the important changes music underwent during the transition from Baroque to Classical. While Baroque and previous music is easily recognized, music types from later periods differ from one another in more-subtle aspects. Another known fact is the rapid change in orchestration during the Classical period, specifically with Beethoven, who doubled the number of certain instruments to the typical orchestra at the time and, as is well accepted, was responsible for the standardization of the orchestra [34,35], giving the music of his time a noticeably different sound. On the side of traditional and popular music, the analysis is similar. Hindu-Raga and Venezuelan music have very particular characteristics. Hindu-Raga music differs from western music in the number of notes it uses, more than the 12 semi-tones defined for western music. Thus, it should not be surprising that Hindu-Raga music appears as an almost separable group from the dodecaphonic music included in the two other groups. When comparing the group Rock-MovieThemes-Chinese with the Venezuelan music, the result is different: the p-values are low, indicating both groups come from different probability distributions for the entropy and the distance to the Zipf’s reference. However, for this pair of groups, the difference does not suffice to make these groups separable.

Looking at the music groups as they were registered in our data, the Univariate and Multivariate performed tests show interesting results that we discuss here. The Univariate ANOVA analysis suggests that the entropy and Zipf’s reference distance have different distributions for academic and traditional music. Entropy showed the least of the differences for academic music (*F* = 1.456), suggesting that using entropy by itself is too weak a parameter to split classes of some academic music periods. Using both parameters, however, offers possibilities for music style classification, even though the overlap between some pairs of groups is strong. This is an expected result, given the independence of the process of classification with respect to the actual musical characteristics of the pieces being classified.

The Wilks tests presented in section 3.4 for the multivariate ANOVA tests show that the entropy and the reference distance of Zipf account for approximately 50% of the location of the mean values for a multivariate distribution of the music groups based on these parameters. While this 50% is not high enough to establish a better separation between music groups, it is certainly enough to accept notable differences in musical genres and styles to be detected by the methods proposed in this study.

### About the evolution of music

Indeed, in Fig 7, it is difficult to distinguish the dominant locations for all types of music. The locations of individual pieces of some types of music are dispersed, and their central location is not easily recognized. However, this does not mean that a piece, properly classified as a specific type of music, does not lie relatively near a certain location that corresponds to the type of music in the space considered. Take, for example, Romantic music (darker blue bubbles) and Baroque music (darker green bubbles). Despite the noticeable dispersion of the bubbles, each group occupies a different volume within the space represented in Fig 7. Both music type clusters are shaped as bows. The one representing Romantic music is located toward the center of the cube, while the Baroque music type cluster is located near the high second-order entropy corner. This aspect of the discussion is important because the standard deviations of the three properties evaluated, presented in Tables 4 and 5, are of the same order as the variation of the averages of the same properties, thus giving the false idea that these clusters are indistinctly dispersed throughout the same space and are, therefore, not separable by the properties suggested here. The reason for why our clusters may be separable while exhibiting high standard deviations, with an apparent full overlap, lies in the clusters’ arched shapes.

Fig 8 shows how each type of music tends to occupy different sectors of the space diversity-entropy. Focusing on academic music, a progression can be seen from the location of Medieval music, located in the sector of high diversity and entropy, to the location of more-recent music, such as Classical and Impressionistic, located at relatively lower specific diversity and entropy. The ordered locations of each type of academic music upon the time parameter suggests that some types of music evolve in a way that can be detected in the mentioned space: *d*, *h*^[1]^, *h*^[2]^.

Traditional Hindu-Raga and traditional Venezuelan music are easily recognizable. There must be properties that make them well defined and different from each other. The fact that Hindu-Raga and Venezuelan music appear to be far from any other style of music in Fig 8 is unsurprising. In fact, it should be taken as a sign of fitness of the space *d*, *h*^[1]^, *h*^[2]^ to represent music differences, and it confirms the prominent distinctions between the profile shapes seen for these types of music in the profiles shown in S2 and S3 Figs.

Considering Fig 9 and S5 Fig, we see that academic music has evolved to produce profiles associated with a lower value of the 2^nd^ order entropy. For academic music, this tendency seems to be sustained from Medieval music to the Impressionistic period. Traditional music and popular music exhibit a 2^nd^ order entropy comparable to the academic music of the 20^th^ Century.

The specific diversity *d*, on the other hand, reveals a slight reduction with time but an increase in the dispersion of this variable starting from Classical music and the Romantic period. This does not allow us to make a clear statement about the sustained tendency of a reduction in specific diversity over time. On the side of traditional and popular music, specific diversity and entropy show less dispersion than their counterpart from academic music at comparable times.

Fig 9 and S5 Fig show the behavior of variables *d*, *h*^[1]^, *h*^[2]^, with each one plotted vs time for academic music. There seems to be a tendency to lower the value of these variables with time. However, the evident increase of the dispersion of these indexes hides the overall change over time of academic music’s entropy. Yet, when the three properties *d*, *h*^[1]^, and *h*^[2]^ form a joint view and time is a parameter, a clustering that migrates from one extreme position to another emerges from the graphs (Figs 7 and 8). This suggests that the combination of the properties *d*, *h*^[1]^, and *h*^[2]^ offers a good basis upon which to build a space where the music style can be recognized.

### Models to represent types of music

The discussion presented above evaluates to what extent these models properly represent the differences among types and styles of music. Although using these methods for some types of music may lead to better results than with other types, we feel we have shown the capacity of the method to capture the most-important characteristics of pieces of music covering a wide range of styles and genres. A numerical model for each of the types of music considered is presented in S1 Table. These models express the structure and the dominant characteristic by means of the relationship of the numbers in any one of the columns of S1 Table. We consider important the fact that the 129 numbers for each type of music properly depict the corresponding type of MIDI music. The reader should recall that these 129 numbers came from degrading a larger set of Fundamental Symbols, which we claim were the best symbolic representation, down to 129 equivalent symbols. Therefore, these 129 symbols represent the most-relevant properties of the structure of the object modeled.

The method can be applied to find a descriptive profile for any group defined. Grouping these pieces by author, we obtained a model for the composers included in this study. Some of these profiles are presented as graphs in S4 Fig. The closeness or separation from one of these composers’ dominant style can be evaluated by computing the Euclidian distance between any pair of profiles, as was explained in section 2.4 (Distance to Zipf’s reference profile).

## Conclusions

Texts produced with music coded by the MIDI synthesizer can be analyzed using symbolic diversity and entropy as variables that can be used to characterize music type and even more-subtle properties, such as style. The inclusion of higher order entropies accentuates the detectable differences between music styles.

We did not use any knowledge of the mechanisms of the MIDI coding process. We started by looking at file texts that seemed to be totally meaningless and indecipherable. By discovering the set of Fundamental Symbols for each music text description, we found several important facts: 1: There is a fundamental symbol set that describes each piece of music. 2: The Fundamental Scale concept, presented in former works, is useful for determining the set of fundamental symbols of machine-coded texts, such as MIDI-music text descriptions. 3: The scale downgrading method proposed allows for comparison of properties of systems of a different nature and at different scales.

By applying the Fundamental Scale Algorithm, we have gone beyond the theoretical considerations about the Minimal Description Length Principle. We built frequency symbol profiles that work as quantitative descriptions for several hundred MIDI pieces. Due to the shapes of these profiles, which are practically unique, these profiles represent a sort of ‘signature’ of the complete polyphonic sound of each musical piece, with most of its subtleties and complexity. After comparing our results for musical pieces according to their music style and period of time, we can affirm that the method works as a consistent procedure to visualize and classify music styles and to quantify differences among them. Due to the arched shapes of the clusters representing each type of music, we did not attempt to create probability fields for each type of music in the space diversity-entropy. However, we foresee the possibility of handling transformations to the shape of the space *d*, *h*^*[1]*^ and *h*^*[2]*^ to achieve the conditions required for a reasonable separation of these clusters, or alternatively, to estimate the probability associating each location in that space with each type of music. However, that would lie within the scope of a future work. For the time being, locating text descriptions in the space specific diversity, entropy and 2nd order entropy and the Euclidian distance between symbol frequency profiles presents a promising tool for classifying MIDI music descriptions, with applications in many research fields, such as quantitative linguistics, pattern recognition, and machine learning.

Music is a reflex of social and cultural likes. We have strived to compare music styles over a quantitative basis. Our results reveal that for all the indexes used to characterize musical genres and styles, there is an increasing dispersion over time. Perhaps this is the image of a society constantly committed to overcoming any cultural barrier, thus making music an expanding phenomenon that grows in any direction of the space we use to observe it. This novel quantitative way of analyzing music might eventually allow us to gain a deeper insight into the musical structures that elicit emotions, illuminating the working of our brains and allowing us to get a better handle on music.

## Supporting information

S1 DataLink (DOCX)

S1 Appendix (DOCX)

S2 Appendix (DOCX)

S1 Table (DOCX)

S1 Fig (DOCX)

S2 Fig (DOCX)

S3 Fig (DOCX)

S4 Fig (DOCX)

S5 Fig (DOCX)

S6 Fig (DOCX)

S7 Fig (DOCX)
