# Supplementary material for: Music viewed by its entropy content: A novel window for comparative analysis
Source: PLoS One. 2017 Oct 17;12(10):e0185757. doi: 10.1371/journal.pone.0185757 (PMC5645004; doi:10.1371/journal.pone.0185757)
Supplement: S1 DataLink — (DOCX) [file pone.0185757.s001.docx]

**S1 Datalink. Music property tree. *MusicNet***

The data resulting from this study can be found in the following URLs:

<http://gfebres.net/Activities/MusicModels/MusicNet.Tree/MusicNet.htm>

<https://figshare.com/articles/Music_Characterizations_MIDI/5435953>

A repository of the MIDI files used in this study can be found in the following URLs:

<https://figshare.com/articles/Music_Medieval_MIDI/5435983>

<https://figshare.com/articles/Music_Reinassance_MIDI/5435992>

<https://figshare.com/articles/Music_Baroque_MIDI/5435995>

<https://figshare.com/articles/Music_Classical_MIDI/5436010>

<https://figshare.com/articles/Music_Romantic_MIDI/5436013>

<https://figshare.com/articles/Music_Impressionistic_MIDI/5436016>

<https://figshare.com/articles/Music_Twentieth_MIDI/5436019>

<https://figshare.com/articles/Music_Traditional_Chinese_MIDI/5436022>

<https://figshare.com/articles/Music_Traditional_HinduRaga_MIDI/5436025>

<https://figshare.com/articles/Music_Traditional_Venezuelan_MIDI/5436106>

<https://figshare.com/articles/Music_Popular_Rock_MIDI/5436031>

<https://figshare.com/articles/Music_Popular_MovieThemes_MIDI/5436034>
