# Supplementary material for: Music viewed by its entropy content: A novel window for comparative analysis
Source: PLoS One. 2017 Oct 17;12(10):e0185757. doi: 10.1371/journal.pone.0185757 (PMC5645004; doi:10.1371/journal.pone.0185757)
Supplement: S1 Appendix — (DOCX) [file pone.0185757.s002.docx]

**S1 Appendix. Math formulation for scale downgrading**

The frequency profile associated to a complex language is a representation of the language. In a language made of *D* different symbols, this representation uses *D* values to describe the language. The graphical representation of these values is useful because it permits to observe an abstract depiction. Depending on the level of detail the observer intends to appreciate, the *D* values may or may not be needed. If for some purpose a rough idea of the profile’s shape is sufficient, a smaller number of values can be used. If on the contrary, the observer needs to detail tiny changes in the profile, a higher density of dots will be required to draw these changes of direction. Changing the number of symbols used to describe a system constitutes a change of the scale of observation of the system; thus we refer to the process of reducing the number of values used to draw the frequency profile as *downgrading the language scale*.


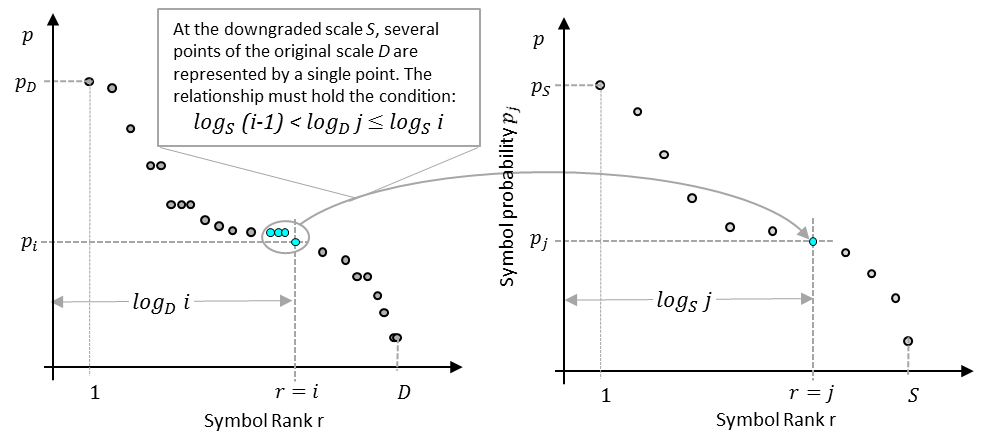


**Fig S1. Graphic representation of a language scale downgrading from scale** $\boldsymbol{D}$ **to scale** $\boldsymbol{S}$ **(**$\boldsymbol{S}\boldsymbol{<}\boldsymbol{D}$**).** The total number of points at scale$D$, representing $D$ symbols on the left graph, are transformed in $S$ points when the language is represented at the scale$S$, as in the right graph.

Consider de language $\boldsymbol{B}$ as a set of $D$ different symbols. If language $\boldsymbol{B}$ is employed to build an $N$-symbol-long system description, then language ***B*** can be specified as the set of $D$ symbols $Y_{i}$ and the probability density function $P(Y_{i})$ which establishes the relative frequencies of appearance of the symbols$f_{i}$ . Thus

|  | $\boldsymbol{B}=$ $\{Y_{1},\ldots, Y_{i}, \ldots, Y_{D} , \boldsymbol{P}(Y_{i})\}$ , | (A) |
| --- | --- | --- |

|  | $P(Y_{i})$= $\frac{f_{i}}{N}$ , $1 \leq i \leq D$ . | (B) |
| --- | --- | --- |

At this point language ***B*** is presented at scale *D*. To include the observation scale of a language as part of the nomenclature, we propose adding a sub-index to the letter representing the language. Then, language ***B*** at some scale *S*, where *1≤ S ≤ D*, would be denoted as ***B****_[S]_*. When the index does not appear, it can be assumed the language is expressed at its original and maximum scale. That is $\boldsymbol{B}=\boldsymbol{B}_{[D]}$. Downgrading a language from scale $D$ to scale $S$ can be performed by pre multiplying vector $\boldsymbol{P}$ with transformation matrix$\boldsymbol{G}$, as indicated below:

|  | $\boldsymbol{P}_{[S]}$= $\boldsymbol{G}_{[S, D]}\cdot\boldsymbol{P}_{[D]}$. | (C) |
| --- | --- | --- |
|  | $\boldsymbol{G}_{[S, D]}$= $\left[ \begin{matrix} \begin{matrix} G_{1,1} & G_{1,2} & \cdots\\ G_{2,1} & \ddots& \cdots\\ \vdots& \vdots& G_{i,j} \end{matrix} & \cdots& \begin{matrix} G_{1,D} \\ \vdots\\ G_{i,D} \end{matrix} \\ \vdots& \ddots& \vdots\\ \begin{matrix} G_{S,1} & \cdots& G_{S,j} \end{matrix} & \cdots& G_{S,D} \end{matrix} \right]$, | (D) |

|  | $G_{i,j}=\left\{ \begin{matrix} 1 if {log}_{D}\left( j-1 \right)\leq{log}_{S}i<{log}_{D}j \\ 0 otherwise \end{matrix} \right., 1\leq j\leq D , 1\leq i\leq S,$ | | (F) |
| --- | --- | --- | --- |
|  | $j=int (S^{{log}_{D} i})$. | (G) | |

This procedure for downgrading the language scale is useful given the frequent requirement of expressing text descriptions at the same scale, which is, using the same number of different symbols.
