# Supplementary material for: Music viewed by its entropy content: A novel window for comparative analysis
Source: PLoS One. 2017 Oct 17;12(10):e0185757. doi: 10.1371/journal.pone.0185757 (PMC5645004; doi:10.1371/journal.pone.0185757)
Supplement: S2 Appendix — (DOCX) [file pone.0185757.s003.docx]

**S2 Appendix. Higher order entropy**

For an ordered symbol frequency distribution, entropy can be used as a general concavity –or convexity– profile index. To obtain an indication about the oscillations of the profile around the middle line represented by the Zipf’s distribution reference line, a new index must generated. We propose the entropy of the distance between the distribution profile and the Zipf’s reference as the new index. Fig B1 illustrates the basis for the definition of this new entropy level.


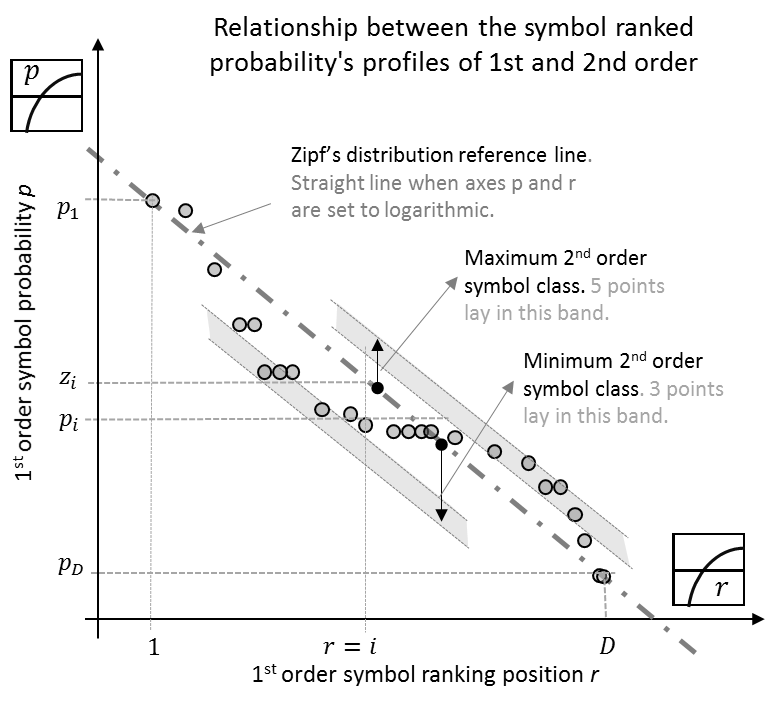


**Fig S2. Typical symbol ranked probability profile with examples of 2^nd^ order symbol bands.** Each dot represent the probability of finding a symbol within all the symbols forming a system description. 1^st^ order symbols are ranked according to their probability of appearance. The most common symbol appears in first place (*r = 1*) and the least frequent symbols appear at the end or tail of the ordered probability distribution representation (*r = D*).

To differentiate these two entropy calculations, we will call this *first order entropy,* or simply, entropy. We refer to the newly created concept as the *second order entropy*. For an ordered probability distribution profile, its first order entropy is sensitive to its overall shape. Since any change of the profile slope needs to run along a wide range of the horizontal axis in order to impact the weighted area calculation, local changes in the profile slope are not effectively captured by the entropy computation. Second order entropy, on the contrary, focuses in the gap between the ordered symbol frequency distribution and the reference Zipf’s distribution, it senses therefore the shape of the oscillations of the symbol probability profile.

To obtain a measure sensitive to small oscillations –or slope changes– we focus the distance $E$between the symbols probability and the imaginary perfect Zipf’s distribution $Z_{i}$that best fits the profile subject to study. The distribution $Z_{i}$ is calculated as follows:

|  | $z_{i}$= $\frac{k}{i^{g}}$ , $g$= $\frac{p_{1}-p_{D}}{D}$ , | (A) (B) |
| --- | --- | --- |

where $g$ is the Zipf’s distribution slope and $k$ is a real number to stablish the starting point on the Zipf line for the first ranked symbol. Notice that $k$ is not necessarily equal to$p_{1}$, as is usually presented. Here the value of $k$have to be adjusted to produce a unitary area under the Zipf’s distribution. The distance$E_{i}$between a symbol probability$p_{i}$and the imaginary Zipf’s distribution $Z_{i}$ is presented as a one-dimensional array.

|  | $\boldsymbol{E}$= $\left[ \begin{matrix} E_{1} \\ E_{2} \\ \vdots\\ E_{D} \end{matrix} \right]$ $= \left[ \begin{aligned} \begin{matrix} p_{1}- z_{1} \\ p_{2}- z_{2} \end{matrix} \\ \vdots\\ p_{D}- z_{D} \end{aligned} \right]$ . | (C) |
| --- | --- | --- |

As depicted in Fig B1, the size of these deviations around the Zipf’s profile can define a new language: the *second order language*. To obtain the 2^nd^ order language we need to define the smallest $\boldsymbol{E}_{min}$ and the largest $\boldsymbol{E}_{max}$and a resolution $q$ to establish the size of the bands to classify the symbols between the values of $\boldsymbol{E}_{min}$and $\boldsymbol{E}_{max}$. After some arithmetic, these band boundaries can synthetized as the one-dimensional array $\boldsymbol{B}$ as:

|  | $\Delta q= \frac{E_{max}- E_{min}}{q}$ , $B_{i}$ = $B_{i-1}$+ $\Delta q$, $B_{1}$ = $E_{min}- \frac{\Delta q}{2}$ | | (D)(E)  (F) | |
| --- | --- | --- | --- | --- |
|  | | $\boldsymbol{B}$= $\left[ \begin{aligned} \begin{matrix} B_{1} \\ B_{2} \end{matrix} \\ \vdots\\ B_{q} \end{aligned} \right]$ | | (G) |

Vectors and distributions associated to an order *u* are represented using a supra-index enclosed by squared brackets. The transition matrix $\boldsymbol{U}$ to relate the distribution at order $u$ with the distribution at order$u-1$, is represented using the supra-index$[u,u-1]$*.* The symbol probability distribution associated to the 2^nd^ order language is represented by the array $\boldsymbol{P}^{[2]}$ and obtained as indicated by Eq. (B4).

|  | $\boldsymbol{P}^{[2]}$= $\boldsymbol{U}^{[1,2]}\cdot\boldsymbol{P}^{[1]}$. | (H) |
| --- | --- | --- |
|  | $\boldsymbol{U}^{\boldsymbol{[}1,2\boldsymbol{]}}$*=* $\left[ \begin{matrix} \begin{matrix} U_{1,1} & U_{1,2} & \cdots\\ U_{2,1} & \ddots& \cdots\\ \vdots& \vdots& U_{i,j} \end{matrix} & \cdots& \begin{matrix} U_{1,D} \\ \vdots\\ U_{i,D} \end{matrix} \\ \vdots& \ddots& \vdots\\ \begin{matrix} U_{q,1} & \cdots& U_{q,j} \end{matrix} & \cdots& U_{q,D} \end{matrix} \right]$ *,* | (I) |
|  | $U_{i,j}= \left\{ \begin{matrix} 1 if B_{i}\leq E_{j}<B_{i+1} \\ 0 else \end{matrix} \right.$ . | (J) |

In general, specifying the desired resolution at some distribution order $q_{u}$ the distribution of any order $u$ can be obtained starting from the preceding order $u-1$ as:

|  | | $\boldsymbol{P}^{[u]}$= $\boldsymbol{U}^{[u-1, u]}\cdot\boldsymbol{P}^{[u-1]}$. | | (K) | | | |
| --- | --- | --- | --- | --- | --- | --- | --- |
|  | | | $\boldsymbol{U}^{[u-1, u]}$= $\left[ \begin{matrix} \begin{matrix} U_{1,1} & U_{1, 2} & \cdots\\ U_{2,1} & \ddots& \cdots\\ \vdots& \vdots& U_{i,j} \end{matrix} & \cdots& \begin{matrix} U_{1, q_{u-1}} \\ \vdots\\ U_{i,q_{u-1}} \end{matrix} \\ \vdots& \ddots& \vdots\\ \begin{matrix} U_{q_{u},1} & \cdots& U_{q_{u},j} \end{matrix} & \cdots& U_{q_{u},q_{u-1}} \end{matrix} \right]$ *,* | | (L) | | |
|  | | $U_{i,j}= \left\{ \begin{matrix} 1 if B_{i} \leq E_{j} < B_{i+1} \\ 0 else \end{matrix} \right.$  *,* | | | | | (M) |
|  | | $\boldsymbol{B}_{u}$= $\left[ \begin{aligned} \begin{matrix} B_{1} \\ B_{2} \end{matrix} \\ \vdots\\ B_{q_{u}} \end{aligned} \right]$ , | | | | (N) | |
|  | $\Delta q_{u}=\frac{E_{max}- E_{min}}{q_{u}}$, ${B_{u}}_{i}$ = ${B_{u}}_{i-1}$+ $\Delta q_{u}$, ${B_{u}}_{1}$= ${E_{u}}_{min}-\frac{\Delta q_{u}}{2}$ . | | | | | (O)( P)  (Q) | |

Finally the $u th$ order entropy can be computed as

|  | $H^{[u]}=-\sum_{i=1}^{q_{u}} B_{i} \log_{q_{u}} B_{i} .$ | (R) |
| --- | --- | --- |
