# Supplementary material for: Music viewed by its entropy content: A novel window for comparative analysis
Source: PLoS One. 2017 Oct 17;12(10):e0185757. doi: 10.1371/journal.pone.0185757 (PMC5645004; doi:10.1371/journal.pone.0185757)
Supplement: S1 Table — (DOCX) [file pone.0185757.s004.docx]

**S1 Table. Music symbol frequency profiles data represented with 128 degrees of freedom**

| **Symbol probability for several types of music. Observation scale = Diversity = 129 Symbols. From r=1 to r=62** | | | | | | | | | | | | |
| --- | --- | --- | --- | --- | --- | --- | --- | --- | --- | --- | --- | --- |
| **rank** | **Medieval** | **Renais.** | **Baroque** | **Classical** | **Romant..** | **Impres.** | **20th Cent.** | **Chinese** | **Hindu Raga** | **Muvie Themes** | **Rock** | **Venez.** |
| 1 | 0.22342 | 0.23910 | 0.28295 | 0.58182 | 0.49362 | 0.56381 | 0.51358 | 0.39492 | 0.23612 | 0.51653 | 0.65791 | 0.66401 |
| 2 | 0.17857 | 0.20810 | 0.19259 | 0.16804 | 0.18914 | 0.10672 | 0.21140 | 0.20506 | 0.11954 | 0.15611 | 0.13894 | 0.10590 |
| 3 | 0.09504 | 0.12754 | 0.12329 | 0.08073 | 0.08830 | 0.05342 | 0.07409 | 0.09071 | 0.07842 | 0.09549 | 0.06132 | 0.05581 |
| 4 | 0.06706 | 0.06938 | 0.09283 | 0.04701 | 0.05730 | 0.03919 | 0.05036 | 0.06755 | 0.07565 | 0.04951 | 0.02577 | 0.03020 |
| 5 | 0.05735 | 0.05924 | 0.07360 | 0.03379 | 0.04213 | 0.03139 | 0.03672 | 0.04753 | 0.07363 | 0.03379 | 0.01394 | 0.01567 |
| 6 | 0.05269 | 0.05114 | 0.06332 | 0.02403 | 0.03147 | 0.02931 | 0.02781 | 0.03410 | 0.06162 | 0.02464 | 0.00722 | 0.01085 |
| 7 | 0.04479 | 0.04213 | 0.04973 | 0.01770 | 0.02652 | 0.02550 | 0.02251 | 0.02775 | 0.05602 | 0.01547 | 0.00485 | 0.00765 |
| 8 | 0.03460 | 0.03639 | 0.03594 | 0.01238 | 0.02228 | 0.02166 | 0.01613 | 0.02104 | 0.04481 | 0.00855 | 0.00405 | 0.00520 |
| 9 | 0.02811 | 0.03104 | 0.02292 | 0.00914 | 0.01674 | 0.01876 | 0.01270 | 0.01702 | 0.02783 | 0.00553 | 0.00357 | 0.00409 |
| 10 | 0.02553 | 0.02403 | 0.01622 | 0.00675 | 0.01132 | 0.01574 | 0.01162 | 0.01453 | 0.01925 | 0.00375 | 0.00306 | 0.00346 |
| 11 | 0.02280 | 0.01917 | 0.01172 | 0.00486 | 0.00826 | 0.01384 | 0.00819 | 0.01174 | 0.01286 | 0.00296 | 0.00272 | 0.00273 |
| 12 | 0.01909 | 0.01614 | 0.00803 | 0.00356 | 0.00455 | 0.01175 | 0.00433 | 0.00950 | 0.01219 | 0.00260 | 0.00243 | 0.00266 |
| 13 | 0.01771 | 0.01242 | 0.00619 | 0.00264 | 0.00206 | 0.01004 | 0.00268 | 0.00812 | 0.01120 | 0.00250 | 0.00234 | 0.00266 |
| 14 | 0.01578 | 0.01013 | 0.00405 | 0.00150 | 0.00080 | 0.00813 | 0.00169 | 0.00709 | 0.01120 | 0.00227 | 0.00227 | 0.00266 |
| 15 | 0.01404 | 0.00871 | 0.00256 | 0.00084 | 0.00041 | 0.00732 | 0.00098 | 0.00639 | 0.01120 | 0.00209 | 0.00206 | 0.00254 |
| 16 | 0.01156 | 0.00636 | 0.00178 | 0.00059 | 0.00030 | 0.00631 | 0.00061 | 0.00571 | 0.01112 | 0.00188 | 0.00193 | 0.00246 |
| 17 | 0.00946 | 0.00452 | 0.00112 | 0.00045 | 0.00025 | 0.00561 | 0.00042 | 0.00475 | 0.01026 | 0.00176 | 0.00190 | 0.00246 |
| 18 | 0.00903 | 0.00374 | 0.00090 | 0.00035 | 0.00023 | 0.00504 | 0.00033 | 0.00367 | 0.00953 | 0.00174 | 0.00188 | 0.00246 |
| 19 | 0.00830 | 0.00316 | 0.00074 | 0.00031 | 0.00020 | 0.00400 | 0.00027 | 0.00287 | 0.00686 | 0.00155 | 0.00178 | 0.00246 |
| 20 | 0.00643 | 0.00257 | 0.00062 | 0.00030 | 0.00019 | 0.00295 | 0.00024 | 0.00163 | 0.00560 | 0.00138 | 0.00170 | 0.00246 |
| 21 | 0.00532 | 0.00206 | 0.00053 | 0.00025 | 0.00017 | 0.00166 | 0.00022 | 0.00086 | 0.00560 | 0.00137 | 0.00157 | 0.00233 |
| 22 | 0.00476 | 0.00153 | 0.00050 | 0.00020 | 0.00016 | 0.00095 | 0.00019 | 0.00071 | 0.00560 | 0.00134 | 0.00147 | 0.00211 |
| 23 | 0.00446 | 0.00117 | 0.00044 | 0.00018 | 0.00015 | 0.00064 | 0.00017 | 0.00062 | 0.00560 | 0.00130 | 0.00147 | 0.00211 |
| 24 | 0.00399 | 0.00105 | 0.00038 | 0.00016 | 0.00014 | 0.00052 | 0.00015 | 0.00057 | 0.00402 | 0.00128 | 0.00138 | 0.00181 |
| 25 | 0.00353 | 0.00094 | 0.00035 | 0.00015 | 0.00013 | 0.00046 | 0.00013 | 0.00054 | 0.00379 | 0.00128 | 0.00129 | 0.00178 |
| 26 | 0.00316 | 0.00087 | 0.00035 | 0.00012 | 0.00012 | 0.00042 | 0.00012 | 0.00050 | 0.00331 | 0.00125 | 0.00123 | 0.00173 |
| 27 | 0.00264 | 0.00081 | 0.00034 | 0.00011 | 0.00011 | 0.00038 | 0.00011 | 0.00047 | 0.00284 | 0.00120 | 0.00122 | 0.00165 |
| 28 | 0.00228 | 0.00073 | 0.00030 | 0.00010 | 0.00011 | 0.00036 | 0.00011 | 0.00044 | 0.00284 | 0.00115 | 0.00119 | 0.00157 |
| 29 | 0.00183 | 0.00065 | 0.00027 | 0.00009 | 0.00011 | 0.00035 | 0.00010 | 0.00042 | 0.00260 | 0.00112 | 0.00117 | 0.00152 |
| 30 | 0.00165 | 0.00058 | 0.00025 | 0.00009 | 0.00010 | 0.00034 | 0.00009 | 0.00040 | 0.00260 | 0.00107 | 0.00117 | 0.00152 |
| 31 | 0.00137 | 0.00056 | 0.00022 | 0.00008 | 0.00010 | 0.00034 | 0.00009 | 0.00039 | 0.00260 | 0.00106 | 0.00111 | 0.00151 |
| 32 | 0.00118 | 0.00053 | 0.00021 | 0.00007 | 0.00009 | 0.00033 | 0.00008 | 0.00036 | 0.00224 | 0.00101 | 0.00110 | 0.00145 |
| 33 | 0.00105 | 0.00049 | 0.00019 | 0.00007 | 0.00009 | 0.00031 | 0.00008 | 0.00035 | 0.00213 | 0.00098 | 0.00102 | 0.00140 |
| 34 | 0.00091 | 0.00046 | 0.00018 | 0.00006 | 0.00008 | 0.00029 | 0.00007 | 0.00033 | 0.00193 | 0.00097 | 0.00100 | 0.00130 |
| 35 | 0.00082 | 0.00043 | 0.00017 | 0.00006 | 0.00008 | 0.00028 | 0.00007 | 0.00032 | 0.00166 | 0.00096 | 0.00099 | 0.00129 |
| 36 | 0.00075 | 0.00041 | 0.00017 | 0.00006 | 0.00008 | 0.00027 | 0.00006 | 0.00031 | 0.00166 | 0.00093 | 0.00098 | 0.00122 |
| 37 | 0.00068 | 0.00038 | 0.00016 | 0.00005 | 0.00008 | 0.00026 | 0.00006 | 0.00029 | 0.00140 | 0.00091 | 0.00097 | 0.00113 |
| 38 | 0.00064 | 0.00036 | 0.00015 | 0.00005 | 0.00007 | 0.00024 | 0.00006 | 0.00028 | 0.00140 | 0.00091 | 0.00096 | 0.00110 |
| 39 | 0.00060 | 0.00035 | 0.00014 | 0.00005 | 0.00007 | 0.00024 | 0.00006 | 0.00027 | 0.00112 | 0.00091 | 0.00096 | 0.00109 |
| 40 | 0.00060 | 0.00035 | 0.00013 | 0.00004 | 0.00007 | 0.00024 | 0.00005 | 0.00026 | 0.00112 | 0.00090 | 0.00096 | 0.00106 |
| 41 | 0.00057 | 0.00033 | 0.00013 | 0.00004 | 0.00006 | 0.00024 | 0.00005 | 0.00025 | 0.00112 | 0.00088 | 0.00096 | 0.00103 |
| 42 | 0.00051 | 0.00030 | 0.00012 | 0.00004 | 0.00006 | 0.00024 | 0.00005 | 0.00024 | 0.00103 | 0.00088 | 0.00091 | 0.00102 |
| 43 | 0.00048 | 0.00029 | 0.00012 | 0.00004 | 0.00006 | 0.00024 | 0.00005 | 0.00023 | 0.00103 | 0.00088 | 0.00088 | 0.00096 |
| 44 | 0.00044 | 0.00028 | 0.00011 | 0.00004 | 0.00006 | 0.00023 | 0.00004 | 0.00022 | 0.00095 | 0.00086 | 0.00084 | 0.00096 |
| 45 | 0.00043 | 0.00028 | 0.00010 | 0.00003 | 0.00006 | 0.00023 | 0.00004 | 0.00021 | 0.00095 | 0.00086 | 0.00082 | 0.00095 |
| 46 | 0.00041 | 0.00027 | 0.00010 | 0.00003 | 0.00006 | 0.00022 | 0.00004 | 0.00020 | 0.00095 | 0.00083 | 0.00079 | 0.00090 |
| 47 | 0.00039 | 0.00027 | 0.00010 | 0.00003 | 0.00005 | 0.00022 | 0.00004 | 0.00020 | 0.00095 | 0.00083 | 0.00078 | 0.00090 |
| 48 | 0.00037 | 0.00025 | 0.00009 | 0.00003 | 0.00005 | 0.00022 | 0.00004 | 0.00019 | 0.00095 | 0.00079 | 0.00075 | 0.00090 |
| 49 | 0.00037 | 0.00025 | 0.00009 | 0.00003 | 0.00005 | 0.00022 | 0.00004 | 0.00018 | 0.00095 | 0.00079 | 0.00068 | 0.00087 |
| 50 | 0.00036 | 0.00025 | 0.00009 | 0.00003 | 0.00005 | 0.00021 | 0.00004 | 0.00018 | 0.00095 | 0.00074 | 0.00064 | 0.00087 |
| 51 | 0.00036 | 0.00024 | 0.00009 | 0.00003 | 0.00005 | 0.00020 | 0.00003 | 0.00018 | 0.00095 | 0.00070 | 0.00064 | 0.00087 |
| 52 | 0.00034 | 0.00024 | 0.00008 | 0.00003 | 0.00005 | 0.00020 | 0.00003 | 0.00018 | 0.00095 | 0.00070 | 0.00063 | 0.00087 |
| 53 | 0.00032 | 0.00024 | 0.00008 | 0.00003 | 0.00004 | 0.00020 | 0.00003 | 0.00018 | 0.00095 | 0.00070 | 0.00062 | 0.00084 |
| 54 | 0.00029 | 0.00022 | 0.00008 | 0.00002 | 0.00004 | 0.00019 | 0.00003 | 0.00017 | 0.00095 | 0.00068 | 0.00061 | 0.00081 |
| 55 | 0.00028 | 0.00020 | 0.00008 | 0.00002 | 0.00004 | 0.00019 | 0.00003 | 0.00016 | 0.00095 | 0.00067 | 0.00059 | 0.00080 |
| 56 | 0.00027 | 0.00019 | 0.00007 | 0.00002 | 0.00004 | 0.00018 | 0.00003 | 0.00016 | 0.00095 | 0.00067 | 0.00059 | 0.00076 |
| 57 | 0.00027 | 0.00019 | 0.00007 | 0.00002 | 0.00004 | 0.00018 | 0.00003 | 0.00016 | 0.00095 | 0.00067 | 0.00058 | 0.00075 |
| 58 | 0.00026 | 0.00019 | 0.00007 | 0.00002 | 0.00004 | 0.00018 | 0.00003 | 0.00016 | 0.00095 | 0.00067 | 0.00058 | 0.00074 |
| 59 | 0.00026 | 0.00018 | 0.00006 | 0.00002 | 0.00004 | 0.00018 | 0.00003 | 0.00016 | 0.00095 | 0.00067 | 0.00054 | 0.00072 |
| 60 | 0.00025 | 0.00018 | 0.00006 | 0.00002 | 0.00004 | 0.00018 | 0.00003 | 0.00016 | 0.00095 | 0.00067 | 0.00053 | 0.00065 |
| 61 | 0.00025 | 0.00018 | 0.00006 | 0.00002 | 0.00004 | 0.00017 | 0.00002 | 0.00015 | 0.00082 | 0.00067 | 0.00052 | 0.00061 |
| 62 | 0.00025 | 0.00017 | 0.00006 | 0.00002 | 0.00003 | 0.00015 | 0.00002 | 0.00015 | 0.00075 | 0.00067 | 0.00051 | 0.00061 |

| **Symbol probability for several types of music. Observation scale = Diversity = 129 Symbols. From r=63 to r=129** | | | | | | | | | | | | |
| --- | --- | --- | --- | --- | --- | --- | --- | --- | --- | --- | --- | --- |
| **rank** | **Medieval** | **Renais.** | **Baroque** | **Classical** | **Romant..** | **Impres.** | **20th Cent.** | **Chinese** | **Hindu Raga** | **Muvie Themes** | **Rock** | **Venez.** |
| 63 | 0.00025 | 0.00016 | 0.00005 | 0.00002 | 0.00003 | 0.00015 | 0.00002 | 0.00014 | 0.00075 | 0.00067 | 0.00051 | 0.00060 |
| 64 | 0.00025 | 0.00016 | 0.00005 | 0.00002 | 0.00003 | 0.00014 | 0.00002 | 0.00013 | 0.00075 | 0.00066 | 0.00049 | 0.00059 |
| 65 | 0.00025 | 0.00016 | 0.00005 | 0.00002 | 0.00003 | 0.00014 | 0.00002 | 0.00012 | 0.00071 | 0.00065 | 0.00047 | 0.00059 |
| 66 | 0.00025 | 0.00015 | 0.00005 | 0.00002 | 0.00003 | 0.00014 | 0.00002 | 0.00012 | 0.00071 | 0.00064 | 0.00044 | 0.00057 |
| 67 | 0.00024 | 0.00014 | 0.00005 | 0.00002 | 0.00003 | 0.00014 | 0.00002 | 0.00012 | 0.00071 | 0.00064 | 0.00043 | 0.00055 |
| 68 | 0.00023 | 0.00014 | 0.00004 | 0.00002 | 0.00003 | 0.00013 | 0.00002 | 0.00012 | 0.00071 | 0.00064 | 0.00043 | 0.00053 |
| 69 | 0.00022 | 0.00014 | 0.00004 | 0.00002 | 0.00003 | 0.00013 | 0.00002 | 0.00012 | 0.00071 | 0.00064 | 0.00043 | 0.00053 |
| 70 | 0.00021 | 0.00014 | 0.00004 | 0.00002 | 0.00003 | 0.00013 | 0.00001 | 0.00012 | 0.00056 | 0.00064 | 0.00043 | 0.00052 |
| 71 | 0.00020 | 0.00013 | 0.00004 | 0.00002 | 0.00003 | 0.00013 | 0.00001 | 0.00011 | 0.00056 | 0.00064 | 0.00042 | 0.00052 |
| 72 | 0.00020 | 0.00013 | 0.00004 | 0.00002 | 0.00002 | 0.00013 | 0.00001 | 0.00010 | 0.00056 | 0.00064 | 0.00041 | 0.00051 |
| 73 | 0.00019 | 0.00013 | 0.00004 | 0.00001 | 0.00002 | 0.00013 | 0.00001 | 0.00009 | 0.00056 | 0.00060 | 0.00041 | 0.00050 |
| 74 | 0.00019 | 0.00013 | 0.00004 | 0.00001 | 0.00002 | 0.00013 | 0.00001 | 0.00009 | 0.00054 | 0.00060 | 0.00041 | 0.00049 |
| 75 | 0.00018 | 0.00013 | 0.00004 | 0.00001 | 0.00002 | 0.00013 | 0.00001 | 0.00009 | 0.00052 | 0.00060 | 0.00040 | 0.00048 |
| 76 | 0.00018 | 0.00013 | 0.00004 | 0.00001 | 0.00002 | 0.00013 | 0.00001 | 0.00009 | 0.00052 | 0.00059 | 0.00040 | 0.00048 |
| 77 | 0.00018 | 0.00012 | 0.00004 | 0.00001 | 0.00002 | 0.00012 | 0.00001 | 0.00009 | 0.00052 | 0.00058 | 0.00040 | 0.00046 |
| 78 | 0.00018 | 0.00012 | 0.00004 | 0.00001 | 0.00002 | 0.00012 | 0.00001 | 0.00009 | 0.00052 | 0.00058 | 0.00040 | 0.00045 |
| 79 | 0.00018 | 0.00012 | 0.00003 | 0.00001 | 0.00002 | 0.00012 | 0.00001 | 0.00009 | 0.00052 | 0.00058 | 0.00040 | 0.00045 |
| 80 | 0.00018 | 0.00012 | 0.00003 | 0.00001 | 0.00002 | 0.00012 | 0.00001 | 0.00009 | 0.00052 | 0.00058 | 0.00040 | 0.00044 |
| 81 | 0.00018 | 0.00011 | 0.00003 | 0.00001 | 0.00002 | 0.00012 | 0.00001 | 0.00009 | 0.00047 | 0.00058 | 0.00039 | 0.00044 |
| 82 | 0.00017 | 0.00010 | 0.00003 | 0.00001 | 0.00001 | 0.00012 | 0.00001 | 0.00009 | 0.00047 | 0.00058 | 0.00039 | 0.00044 |
| 83 | 0.00016 | 0.00010 | 0.00003 | 0.00001 | 0.00001 | 0.00012 | 0.00001 | 0.00009 | 0.00047 | 0.00058 | 0.00039 | 0.00033 |
| 84 | 0.00016 | 0.00010 | 0.00003 | 0.00001 | 0.00001 | 0.00012 | 0.00001 | 0.00009 | 0.00047 | 0.00057 | 0.00039 | 0.00033 |
| 85 | 0.00016 | 0.00009 | 0.00003 | 0.00001 | 0.00001 | 0.00012 | 0.00001 | 0.00009 | 0.00047 | 0.00057 | 0.00039 | 0.00033 |
| 86 | 0.00016 | 0.00009 | 0.00003 | 0.00001 | 0.00001 | 0.00012 | 0.00001 | 0.00009 | 0.00047 | 0.00057 | 0.00039 | 0.00033 |
| 87 | 0.00015 | 0.00009 | 0.00003 | 0.00001 | 0.00001 | 0.00012 | 0.00001 | 0.00009 | 0.00047 | 0.00057 | 0.00039 | 0.00032 |
| 88 | 0.00015 | 0.00008 | 0.00003 | 0.00001 | 0.00001 | 0.00012 | 0.00001 | 0.00009 | 0.00047 | 0.00056 | 0.00038 | 0.00032 |
| 89 | 0.00014 | 0.00008 | 0.00002 | 0.00001 | 0.00001 | 0.00012 | 0.00001 | 0.00008 | 0.00047 | 0.00055 | 0.00038 | 0.00032 |
| 90 | 0.00014 | 0.00008 | 0.00002 | 0.00001 | 0.00001 | 0.00012 | 0.00001 | 0.00008 | 0.00047 | 0.00055 | 0.00031 | 0.00032 |
| 91 | 0.00014 | 0.00007 | 0.00002 | 0.00001 | 0.00001 | 0.00012 | 0.00001 | 0.00008 | 0.00047 | 0.00053 | 0.00027 | 0.00032 |
| 92 | 0.00014 | 0.00007 | 0.00002 | 0.00001 | 0.00001 | 0.00012 | 0.00001 | 0.00008 | 0.00047 | 0.00053 | 0.00027 | 0.00032 |
| 93 | 0.00013 | 0.00007 | 0.00002 | 0.00001 | 0.00001 | 0.00012 | 0.00001 | 0.00008 | 0.00047 | 0.00053 | 0.00027 | 0.00031 |
| 94 | 0.00012 | 0.00006 | 0.00002 | 0.00001 | 0.00001 | 0.00012 | 0.00000 | 0.00008 | 0.00047 | 0.00051 | 0.00027 | 0.00031 |
| 95 | 0.00012 | 0.00006 | 0.00002 | 0.00001 | 0.00001 | 0.00011 | 0.00000 | 0.00008 | 0.00047 | 0.00047 | 0.00027 | 0.00031 |
| 96 | 0.00012 | 0.00006 | 0.00002 | 0.00001 | 0.00001 | 0.00009 | 0.00000 | 0.00008 | 0.00047 | 0.00047 | 0.00027 | 0.00031 |
| 97 | 0.00012 | 0.00006 | 0.00002 | 0.00001 | 0.00001 | 0.00009 | 0.00000 | 0.00008 | 0.00047 | 0.00046 | 0.00027 | 0.00030 |
| 98 | 0.00011 | 0.00006 | 0.00002 | 0.00001 | 0.00001 | 0.00009 | 0.00000 | 0.00008 | 0.00047 | 0.00045 | 0.00027 | 0.00030 |
| 99 | 0.00010 | 0.00006 | 0.00002 | 0.00001 | 0.00001 | 0.00009 | 0.00000 | 0.00008 | 0.00047 | 0.00045 | 0.00027 | 0.00030 |
| 100 | 0.00010 | 0.00006 | 0.00002 | 0.00001 | 0.00001 | 0.00009 | 0.00000 | 0.00008 | 0.00047 | 0.00045 | 0.00027 | 0.00030 |
| 101 | 0.00010 | 0.00006 | 0.00002 | 0.00001 | 0.00001 | 0.00007 | 0.00000 | 0.00008 | 0.00047 | 0.00045 | 0.00026 | 0.00030 |
| 102 | 0.00010 | 0.00006 | 0.00002 | 0.00001 | 0.00001 | 0.00007 | 0.00000 | 0.00008 | 0.00047 | 0.00045 | 0.00026 | 0.00030 |
| 103 | 0.00010 | 0.00006 | 0.00002 | 0.00001 | 0.00001 | 0.00006 | 0.00000 | 0.00008 | 0.00028 | 0.00045 | 0.00026 | 0.00030 |
| 104 | 0.00009 | 0.00006 | 0.00002 | 0.00001 | 0.00001 | 0.00006 | 0.00000 | 0.00008 | 0.00028 | 0.00044 | 0.00026 | 0.00030 |
| 105 | 0.00009 | 0.00005 | 0.00002 | 0.00001 | 0.00001 | 0.00006 | 0.00000 | 0.00008 | 0.00028 | 0.00044 | 0.00026 | 0.00030 |
| 106 | 0.00009 | 0.00005 | 0.00002 | 0.00001 | 0.00001 | 0.00006 | 0.00000 | 0.00008 | 0.00028 | 0.00043 | 0.00026 | 0.00030 |
| 107 | 0.00009 | 0.00005 | 0.00002 | 0.00000 | 0.00001 | 0.00006 | 0.00000 | 0.00008 | 0.00028 | 0.00039 | 0.00026 | 0.00030 |
| 108 | 0.00009 | 0.00005 | 0.00002 | 0.00000 | 0.00001 | 0.00006 | 0.00000 | 0.00008 | 0.00028 | 0.00039 | 0.00026 | 0.00030 |
| 109 | 0.00009 | 0.00005 | 0.00002 | 0.00000 | 0.00001 | 0.00006 | 0.00000 | 0.00008 | 0.00028 | 0.00038 | 0.00026 | 0.00030 |
| 110 | 0.00009 | 0.00004 | 0.00001 | 0.00000 | 0.00001 | 0.00006 | 0.00000 | 0.00008 | 0.00028 | 0.00037 | 0.00026 | 0.00030 |
| 111 | 0.00009 | 0.00003 | 0.00001 | 0.00000 | 0.00001 | 0.00006 | 0.00000 | 0.00008 | 0.00028 | 0.00037 | 0.00026 | 0.00030 |
| 112 | 0.00009 | 0.00003 | 0.00001 | 0.00000 | 0.00001 | 0.00006 | 0.00000 | 0.00008 | 0.00028 | 0.00037 | 0.00026 | 0.00030 |
| 113 | 0.00008 | 0.00003 | 0.00001 | 0.00000 | 0.00001 | 0.00006 | 0.00000 | 0.00008 | 0.00024 | 0.00037 | 0.00025 | 0.00030 |
| 114 | 0.00008 | 0.00003 | 0.00001 | 0.00000 | 0.00001 | 0.00006 | 0.00000 | 0.00008 | 0.00024 | 0.00037 | 0.00022 | 0.00029 |
| 115 | 0.00008 | 0.00003 | 0.00001 | 0.00000 | 0.00001 | 0.00006 | 0.00000 | 0.00008 | 0.00024 | 0.00035 | 0.00021 | 0.00026 |
| 116 | 0.00008 | 0.00003 | 0.00001 | 0.00000 | 0.00001 | 0.00006 | 0.00000 | 0.00008 | 0.00024 | 0.00034 | 0.00021 | 0.00025 |
| 117 | 0.00008 | 0.00003 | 0.00001 | 0.00000 | 0.00001 | 0.00006 | 0.00000 | 0.00008 | 0.00024 | 0.00034 | 0.00020 | 0.00017 |
| 118 | 0.00008 | 0.00003 | 0.00001 | 0.00000 | 0.00000 | 0.00006 | 0.00000 | 0.00008 | 0.00024 | 0.00032 | 0.00020 | 0.00017 |
| 119 | 0.00008 | 0.00003 | 0.00001 | 0.00000 | 0.00000 | 0.00006 | 0.00000 | 0.00008 | 0.00024 | 0.00032 | 0.00020 | 0.00016 |
| 120 | 0.00008 | 0.00003 | 0.00001 | 0.00000 | 0.00000 | 0.00006 | 0.00000 | 0.00007 | 0.00024 | 0.00032 | 0.00019 | 0.00016 |
| 121 | 0.00007 | 0.00003 | 0.00001 | 0.00000 | 0.00000 | 0.00006 | 0.00000 | 0.00004 | 0.00024 | 0.00031 | 0.00019 | 0.00016 |
| 122 | 0.00005 | 0.00003 | 0.00001 | 0.00000 | 0.00000 | 0.00006 | 0.00000 | 0.00004 | 0.00024 | 0.00029 | 0.00014 | 0.00015 |
| 123 | 0.00005 | 0.00003 | 0.00001 | 0.00000 | 0.00000 | 0.00006 | 0.00000 | 0.00004 | 0.00024 | 0.00028 | 0.00014 | 0.00015 |
| 124 | 0.00005 | 0.00003 | 0.00001 | 0.00000 | 0.00000 | 0.00005 | 0.00000 | 0.00004 | 0.00024 | 0.00026 | 0.00013 | 0.00015 |
| 125 | 0.00005 | 0.00003 | 0.00001 | 0.00000 | 0.00000 | 0.00003 | 0.00000 | 0.00004 | 0.00024 | 0.00024 | 0.00013 | 0.00015 |
| 126 | 0.00004 | 0.00003 | 0.00001 | 0.00000 | 0.00000 | 0.00003 | 0.00000 | 0.00004 | 0.00024 | 0.00022 | 0.00013 | 0.00015 |
| 127 | 0.00004 | 0.00002 | 0.00001 | 0.00000 | 0.00000 | 0.00003 | 0.00000 | 0.00004 | 0.00024 | 0.00019 | 0.00013 | 0.00015 |
| 128 | 0.00004 | 0.00002 | 0.00001 | 0.00000 | 0.00000 | 0.00003 | 0.00000 | 0.00004 | 0.00024 | 0.00019 | 0.00013 | 0.00015 |
| 129 | 0.00004 | 0.00002 | 0.00000 | 0.00000 | 0.00000 | 0.00003 | 0.00000 | 0.00004 | 0.00024 | 0.00019 | 0.00013 | 0.00015 |
