# Supplementary material for: Music viewed by its entropy content: A novel window for comparative analysis
Source: PLoS One. 2017 Oct 17;12(10):e0185757. doi: 10.1371/journal.pone.0185757 (PMC5645004; doi:10.1371/journal.pone.0185757)
Supplement: S1 Fig — (DOCX) [file pone.0185757.s005.docx]

**S1 Fig. Information profiles of some selected music**

**
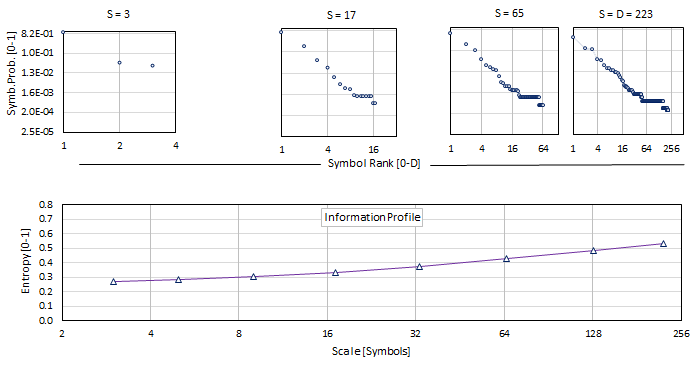
**

**S1 Fig A. Information profile for Beethoven’s 9^th^ Symphony 4^th^ Movement.**

**.**

**
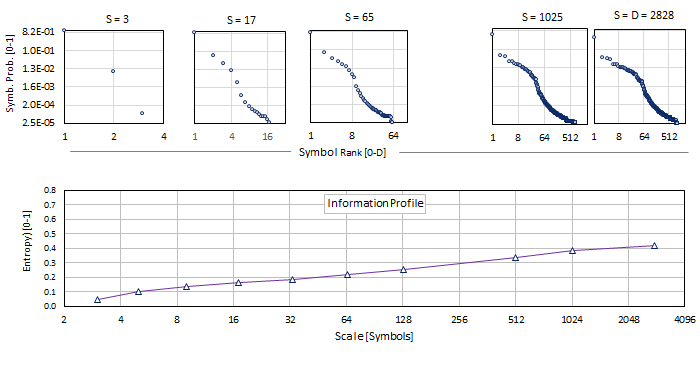
**

**S1 Fig B. Information profile for ValsVenezolanoNro3, Natalia, LAURO.Antonio.**
