# Supplementary material for: Music viewed by its entropy content: A novel window for comparative analysis
Source: PLoS One. 2017 Oct 17;12(10):e0185757. doi: 10.1371/journal.pone.0185757 (PMC5645004; doi:10.1371/journal.pone.0185757)
Supplement: S2 Fig — (DOCX) [file pone.0185757.s006.docx]

**S2 Fig. Symbol ranked frequency profiles for 12 styles of music**

**
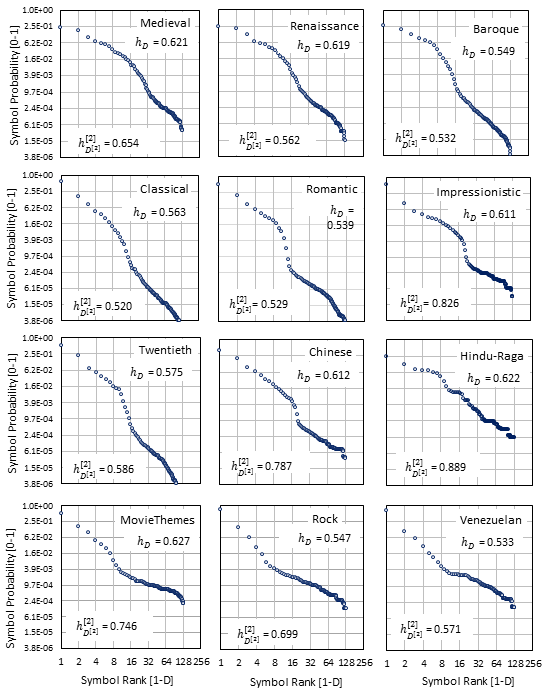
**

**S2 Fig. Symbol frequency profiles for 12 styles of music.**
