# Supplementary material for: Music viewed by its entropy content: A novel window for comparative analysis
Source: PLoS One. 2017 Oct 17;12(10):e0185757. doi: 10.1371/journal.pone.0185757 (PMC5645004; doi:10.1371/journal.pone.0185757)
Supplement: S4 Fig — (DOCX) [file pone.0185757.s008.docx]

**S4 Fig. Symbol frequency profiles for composers**

**
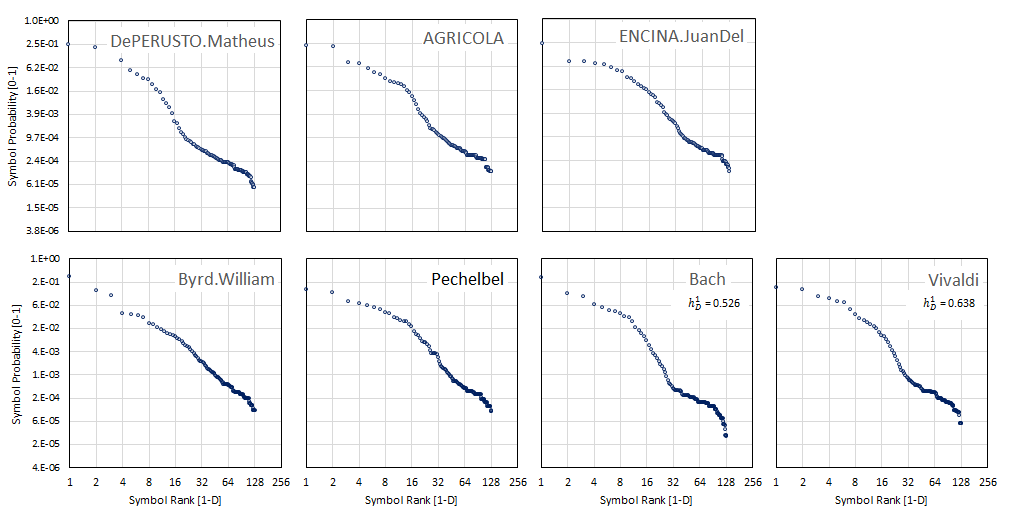
**

**S4 Fig A. Symbol frequency profiles for composers of Baroque and previous periods.**

**
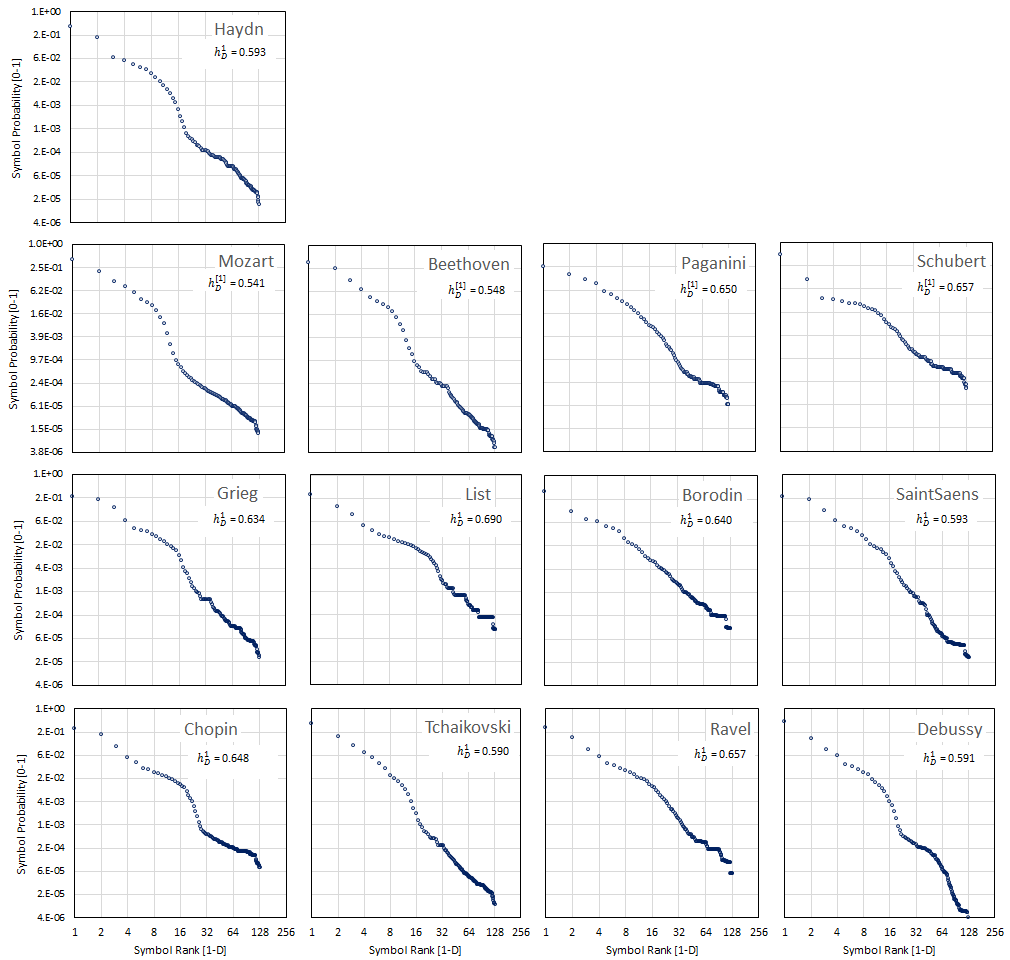
**

**S4 Fig B. Symbol frequency profiles for composers of Classical and following periods.**

**
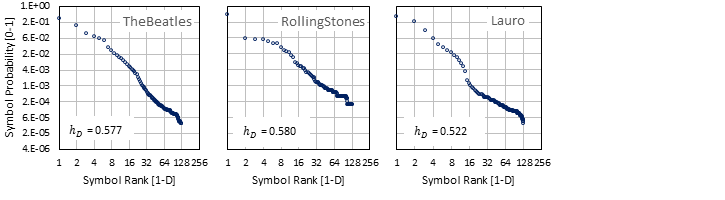
**

**S4 Fig C. Symbol frequency profiles for composers of traditional and popular styles.**
