# Supplementary material for: Music viewed by its entropy content: A novel window for comparative analysis
Source: PLoS One. 2017 Oct 17;12(10):e0185757. doi: 10.1371/journal.pone.0185757 (PMC5645004; doi:10.1371/journal.pone.0185757)
Supplement: S5 Fig — (DOCX) [file pone.0185757.s009.docx]

**S5 Fig. Change of specific diversity and entropy over time**

**
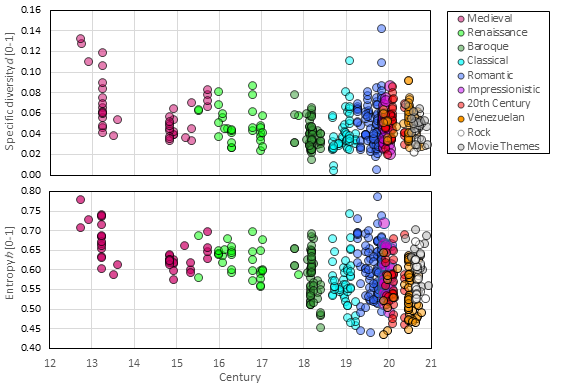
**

**S5 Fig. Change of specific diversity and entropy over last centuries for genres and styles of music.**
