# Supplementary material for: Music viewed by its entropy content: A novel window for comparative analysis
Source: PLoS One. 2017 Oct 17;12(10):e0185757. doi: 10.1371/journal.pone.0185757 (PMC5645004; doi:10.1371/journal.pone.0185757)
Supplement: S6 Fig — (DOCX) [file pone.0185757.s010.docx]

# S6 Fig. Music styles by composer, in the space *(*specific diversity, entropy, 2^nd^ order entropy), (*d, h^[1]^ , h^[2]^* )

**
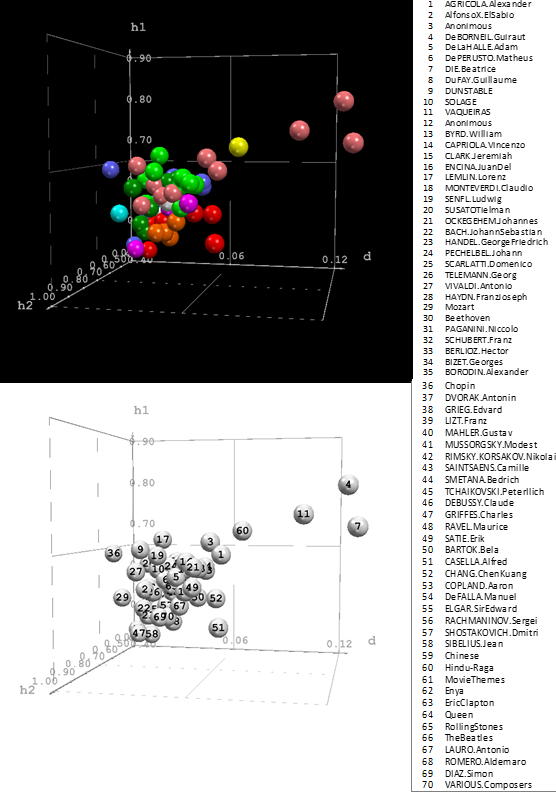
**

**S6 Fig A. A perspective of composers’ locations in the space (*d, h^[1]^ , h^[2]^* ).**

**
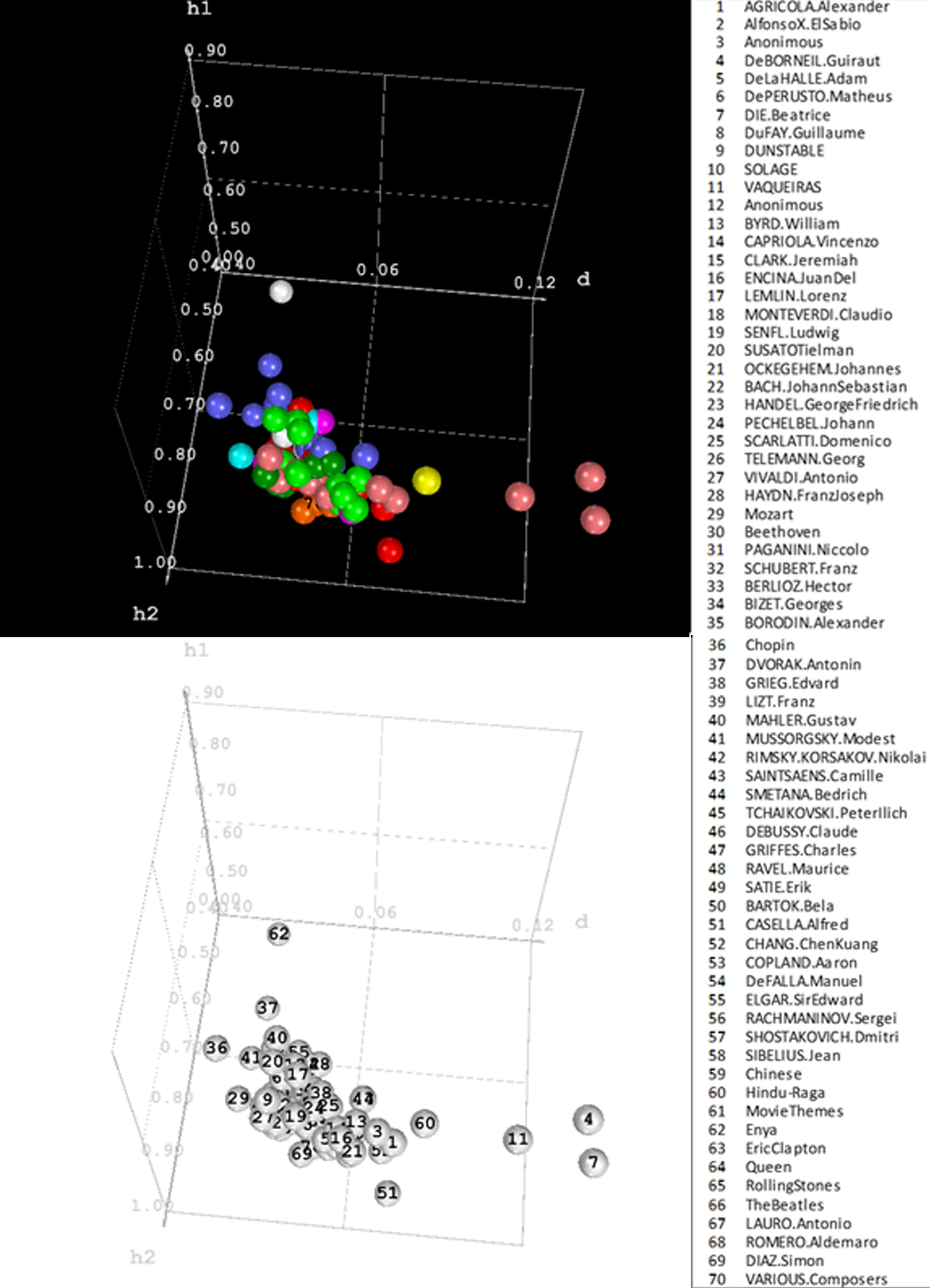
**

**S6 Fig B. A perspective of composers’ locations in the space (*d, h^[1]^ , h^[2]^* ).**
