# Supplementary material for: Music viewed by its entropy content: A novel window for comparative analysis
Source: PLoS One. 2017 Oct 17;12(10):e0185757. doi: 10.1371/journal.pone.0185757 (PMC5645004; doi:10.1371/journal.pone.0185757)
Supplement: S7 Fig — (DOCX) [file pone.0185757.s011.docx]

# S7 Fig. Location of Classical composers represented in the space specific diversity, entropy, 2^nd^ order entropy (*d, h^[1]^ , h^[2]^*).

#
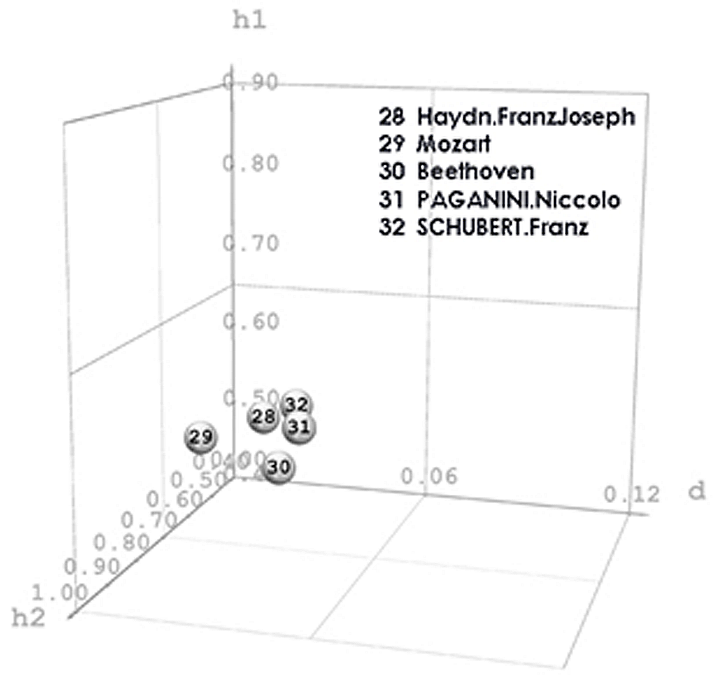

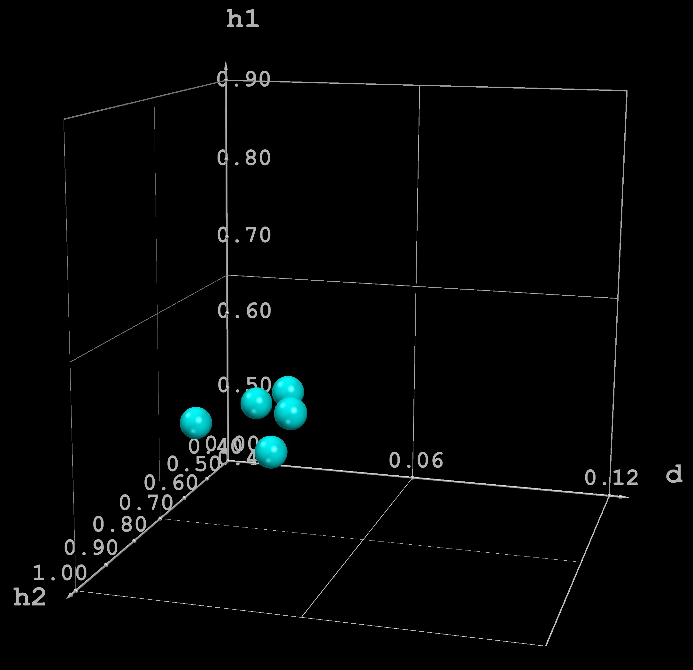


**S7 Fig. A perspective of Classical composers’ locations in the space (*d, h^[1]^ , h^[2]^* ).** Each bubble represents the dominant location of the music of a composer.
